# Effects of gut microbiota interventions on patients with schizophrenia: a systematic review and meta-analysis

**DOI:** 10.3389/fmicb.2025.1681559

**Published:** 2025-11-06

**Authors:** Nianhua Ye, Xin Song, Jing Yu, Xiaolei Bao, Minghua Ye, Lisheng Jiang

**Affiliations:** 1 College of Traditional Chinese Medicine, Jiangxi University of Chinese Medicine, Nanchang, Jiangxi, China; 2 College of Humanities, Jiangxi University of Chinese Medicine, Nanchang, Jiangxi, China; 3 College of Chinese Classics Studies, Beijing University of Chinese Medicine, Beijing, China

**Keywords:** gut microbiota, probiotics, schizophrenia, effects, meta-analysis

## Abstract

**Introduction:**

Schizophrenia (SCH) is a chronic psychiatric disorder characterized by disturbances in thought, emotion, perception, and behavior. Although gut microbiota interventions (e.g., probiotics, prebiotics, synbiotics, dietary modifications and fecal microbiota transplantation) have been widely applied in the treatment of SCH, the most effective intervention strategy remains uncertain.

**Methods:**

By searching four databases, only randomized controlled trials (RCTs) were included to examine the impacts of gut microbiota interventions on SCH. The Cochrane risk-of-bias tool for randomized trials (RoB 2.0) was employed to assess the methodological quality of the included studies, RevMan5.4 was used for the meta-analysis, Stata 18 was used for sensitivity analysis, Engauge Digitizer was used to convert pictures to numbers and GRADEPro3.6 was used to grade the evidence quality.

**Results:**

This study incorporated RCTs published from the earliest records up to December 2024. A total of 10 RCTs, encompassing 585 participants, were analyzed. The meta-analysis demonstrated that interventions primarily utilizing probiotics to modulate gut microbiota significantly lowered the total Positive and Negative Syndrome Scale (PANSS) scores among patients (*p* = 0.001). Furthermore, substantial improvements were observed across multiple metabolic parameters: fasting blood sugar, triglycerides, total cholesterol, homeostasis model assessment of insulin resistance, and quantitative insulin sensitivity check index (all *p* < 0.05). While no significant effects were observed on high-density lipoprotein cholesterol, low-density lipoprotein cholesterol, body weight, body mass index, and insulin.

**Conclusion:**

This meta-analysis suggests that auxiliary probiotic interventions hold promise as an adjunctive therapy for schizophrenia, potentially yielding benefits in psychopathological, metabolic, and physiological domains. However, the current evidence remains inconclusive due to the limited number of studies, small sample sizes, and methodological variations. Firm therapeutic recommendations cannot be made at this time. The findings underscore the need for more robust, large-scale, and rigorously designed randomized controlled trials to definitively establish the efficacy and optimal protocols of auxiliary probiotic supplementation for SCH.

**Systematic review registration:**

https://www.crd.york.ac.uk/PROSPERO, CRD 420250652507.

## 1 Introduction

Schizophrenia (SCH), a chronic psychiatric disorder, is characterized by disturbances in thought, emotion, perception, and behavior, with core clinical features comprise positive symptoms, negative symptoms, and cognitive impairment (Insel, 2010; Matcovitch-Natan et al., 2016). According to the data report of WHO, the global prevalence of SCH is approximately 0.7%, affecting 45 to 50 million individuals worldwide. This disorder typically occurs during late adolescence to early adulthood (Insel, 2010; Sommer et al., 2020), with a relapse rate reaching 80% and significant social functional disability (Fakorede et al., 2022). Current clinical management follows multimodal intervention principles, primarily involving second-generation antipsychotics (e.g., risperidone, olanzapine) in combination with cognitive behavioral therapy and social skills training. However, approximately 30% of patients demonstrate treatment resistance to conventional pharmacotherapy, while drug-related adverse effects, such as metabolic syndrome, significantly compromise therapeutic efficacy (Howes et al., 2021), thereby driving the ongoing exploration of novel interventions.

Recent advances in microbiome research have elucidated the role of the gut–brain axis in modulating neuroinflammation and neurotransmitter metabolism, offering a novel therapeutic paradigm for schizophrenia (Sarkar et al., 2018). Although even some antipsychotics such as amisulpride may partially exert their effects through microbial modulation (Zheng et al., 2022), their use is often limited by adverse metabolic and cognitive effects (Zheng et al., 2019; Nita et al., 2022). Consequently, gut microbiota interventions—including probiotics, prebiotics, synbiotics, fecal microbiota transplantation (FMT), and dietary changes—have been increasingly investigated as adjunctive strategies to improve psychopathological and metabolic outcomes in schizophrenia. Studies suggest that certain interventions show promise: for instance, *Bifidobacterium breve* A-1 may alleviate affective symptoms (Okubo et al., 2019; Yamamura et al., 2021), while FMT could restore microbial ecology (Singh et al., 2024), and dietary fiber may enhance butyrate production (Marx et al., 2020). Probiotics have also been found to attenuate antipsychotic-induced weight gain and improve cognitive function in some trials (Kao et al., 2018; Yang et al., 2021; Xavier et al., 2022; Haghighi et al., 2023; Frileux et al., 2024). Prebiotics may selectively enrich butyrate-producing bacteria, potentially mitigating somatic comorbidities (Buchanan et al., 2024).

However, the available evidence remains inconsistent and heterogeneous. While several randomized controlled trials (RCTs) report benefits in symptoms and cognition (Genedi et al., 2019; Borkent et al., 2024), others show no significant clinical improvement correlating with microbial changes (Mörkl et al., 2020), and few demonstrate symptom-specific efficacy (Forth et al., 2023). Previous meta-analyses have begun to synthesize this literature, yet limitations such as narrow intervention types, small sample sizes, and variable outcome measures preclude definitive conclusions. This systematic review therefore aims to consolidate current RCT evidence, explicitly addressing the inconsistency in outcomes and providing an updated and comprehensive evaluation of the efficacy of gut microbiota-focused interventions, including probiotics, prebiotics, synbiotics, dietary modifications and fecal microbiota transplantation, in schizophrenia.

Meta-analysis is a systematic review methodology. It enables the quantitative synthesis of data from multiple independent studies to enhance statistical power, reduce bias, and reliably evaluate intervention efficacy (Egger et al., 1997). In order to quantitatively synthesize the burgeoning yet inconsistent evidence, this study conducts a meta-analysis of gut microbiota-targeted interventions in SCH patients, which was designed based on the PICOT framework. Firstly, the population is patients with schizophrenia (SCH) of any etiology; secondly, the intervention group adopted gut microbiota-targeted interventions (including probiotics, prebiotics, synbiotics, fermented food, or specific dietary regulation); thirdly, the comparison group adopted the placebo or conventional treatment (e.g., antipsychotics like olanzapine or general dietary advice); fourthly, the outcomes including the primary outcome and the secondary outcomes, the primary outcome was the change in psychopathological symptoms measured by the total score of the Positive and Negative Syndrome Scale, and the key secondary outcomes included Brief Psychiatric Rating Scale (BPRS) score, fasting blood sugar (FBS) level, insulin (INS), triglyceride (TG), total cholesterol (TC), high-density lipoprotein cholesterol (HDL-cholesterol) levels, low-density lipoprotein cholesterol (LDL-cholesterol) levels, homeostasis model assessment of insulin resistance (HOMA-IR), quantitative insulin sensitivity check index (QUICKI), body weight (BW), and body mass index (BMI); finally, the time duration of intervention the included RCTs is from 6 weeks (Mujahid et al., 2022) to 6 months (Sevillano-Jiménez et al., 2022). Therefore, this study aims to: (1) evaluate the clinical efficacy of probiotics, synbiotics, and nutritional interventions on psychopathological manifestations, metabolic parameters, and physiological; (2) explore potential mechanisms underlying microbiota-mediated clinical improvements; (3) elucidate consistencies and discrepancies across the existing evidence. These findings are expected to furnish evidence-based directives for personalized therapeutic approaches in SCH, concurrently delineating pivotal knowledge deficiencies to inform subsequent research priorities.

## 2 Materials and methods

This study adhered to the standard requirements of the Preferred Reporting Items for Systematic Reviews and Meta-analysis (PRISMA), which are used for reporting meta-analyses. And the PRISMA checklist is presented in Supplementary Table S1. The systematic review protocol has been registered in the International Prospective Register of Systematic Reviews (PROSPERO), and the registration number is CRD 420250652507.

### 2.1 Search strategy

We carried out a search for full-text original research articles on gut microbiota interventions for schizophrenia in PubMed, Web of Science, the Cochrane Library, EMBASE, and gray literature sources. The search covered the period from the establishment of each database until December 2024. The strategy employed key terms such as “probiotic*,” “prebiotic*,” “synbiotic*,” “lactobacillus,” “bifidobacterium,” “fecal microbiota transplantation,” “diet regulation,” “fermented food,” “schizophreni*.” We used theme words and free words combined to create database-specific search plans, which were optimized during the search. The search language was English. We also checked references of related reviews and system evaluations to find possible eligible studies. Supplementary searches were performed on ClinicalTrials.gov to identify gray literature; however, no additional data suitable for inclusion were identified. And the detailed strategies of search are provided in Supplementary Table S2.

### 2.2 Inclusion criteria

The inclusion criteria were established according to the PICOS framework.

Population: Patients with SCH of any etiology.
Intervention: Patients in the experimental group received probiotics, prebiotics, synbiotics, fermented food, FMT or dietary regulation as interventions for SCH.
Comparison: Patients in the control group underwent placebo or conventional treatment (olanzapine or dietary advice).
Outcomes: The outcomes included psychopathological symptoms, metabolic, and physiological indexes/tests for individuals with SCH. The primary outcome was the total a core of PANSS, and the key secondary outcomes included BPRS score, and the level of FBS, INS, TG, TC, HDL-cholesterol, LDL-cholesterol, HOMA-IR, QUICKI, BW, and BMI.
Study type: Published RCTs on the treatment of SCH. And these studies are peer-reviewed and written in English.

### 2.3 Exclusion criteria

Exclusion criteria comprised: (1) studies with inadequate or unavailable data; (2) non-RTC designs, including animal research, review articles, conference proceedings, protocols, case reports, commentaries, or letters; (3) duplicate publications; and (4) studies whose full-text was inaccessible.

### 2.4 Data extraction

EndNoteTM^20^ was employed to manage and remove duplicate studies. Following this, two reviewers (XS and JY) independently screened the titles, abstracts and keywords. Subsequently, all potential research papers were thoroughly reviewed to exclude those failing to meet the inclusion criteria. If there were any disagreements among two reviewers, it should be settled by consulting with a third reviewer (XB).

Two investigators (XS and MY) independently performed data extraction utilizing a standardized collection template. This template was structured into four sections: (1) fundamental attributes of the included trials, encompassing details such as the primary author, publication country and year, randomization procedures, and allocation concealment techniques; (2) participant demographics, detailing sample size, sex distribution, and age range; (3) intervention characteristics, including treatment measures, such as the details of the gut microbiota intervention, and duration; (4) outcomes: the average values and standard deviations before and after treatment. If the average values were not provided in the study, we would estimate the average values and standard deviations based on the median, range, interquartile range and/or 95% confidence interval range (Luo et al., 2016; Wan et al., 2014; Liu et al., 2017). The extraction data was collected in Supplementary Table S3. Upon completion of data extraction, the two reviewers swapped their completed forms for cross-checking. Any discrepancies identified were first addressed through discussion, where they explained their rationales. If consensus remained elusive, the third reviewer (LJ) would be consulted to arbitrate and facilitate a resolution.

### 2.5 Risk of bias in individual studies

A revised Cochrane risk-of-bias tool for randomized trials (RoB 2.0) was employed to evaluate the risk of bias in the included studies (Higgins et al., 2011; Sterne et al., 2019). The risk-of-bias table incorporated bias stemming from the randomization process, bias resulting from deviations from the intended interventions, bias associated with missing data, bias related to outcome measurement, and bias arising from the selection of reported results. Each trial was evaluated as either high risk, some concerns, or low risk. Two investigators (XS and XB) independently evaluated the risk of bias in RCTs applying this tool. In instances where discrepancies arose, the two reviewers initially engaged in discussions to explore the rationale behind their divergent evaluations and expressed their viewpoints. If disagreement still persisted through discussion, the third researcher (MY) was enlisted to offer an additionally objective perspective and facilitate mediation to gain consensus.

### 2.6 Data analysis

The quality assessment of RCT articles was conducted utilizing Cochrane RevMan 5.4. For studies employing gut microbiota interventions, where all outcome variables were continuous and reported as mean ± standard deviation. Continuous variables in the study were represented as either the mean difference (MD = the absolute difference between the means of the two group’s data, namely, the difference between the intervention group’s mean and the control group’s mean, calculated based on the same scale) or the standardized mean difference (SMD = the mean difference between the groups divided by the standard deviation of the subjects’ results, used to combine trial data for different scales), reported with 95% confidence intervals (CI). And the differences between the baseline and post-treatment means and standard deviations (SDs) were used to conduct meta-analysis. MD were determined by subtracting the baseline value from the post-treatment value. When studies did not provide the SDs of the changes in outcomes, these values were estimated using a correlation coefficient (*r*) of 0.5 and the equation:

$${\mathrm{SD}}_{\text{change}}=\sqrt{{\mathrm{SD}}_{\text{baseline}}^{2}+{\mathrm{SD}}_{\text{final}}^{2}-(2\times r\times {\mathrm{SD}}_{\text{baseline}}\times {\mathrm{SD}}_{\text{final}})}$$

Then, *I*^2^ statistic is the criterion used in this study to assess heterogeneity, when *I*^2^ > 50%, a random effects model (REM) was used for analysis; when *I*^2^ < 50%, a fixed effects model (FEM) was used.

Sensitivity analysis was conducted by Stata 18.0, we excluded the original studies one by one and combined the effect sizes of the remaining studies.

Engauge Digitizer was used to convert pictures to numbers, if the variables are not reported in numerical terms.

The certainty of evidence in this study was evaluated using the Grading of Recommendations Assessment, Development and Evaluation (GRADE) framework (Guyatt et al., 2008), which rates each by considering factors such as study design, risk of bias, consistency, directness, precision, and publication bias. The quality of evidence for each outcome is categorized as high, moderate, low, or very low (Supplementary Table S4).

## 3 Results

### 3.1 Study, identification and selection

**Figure 1:**
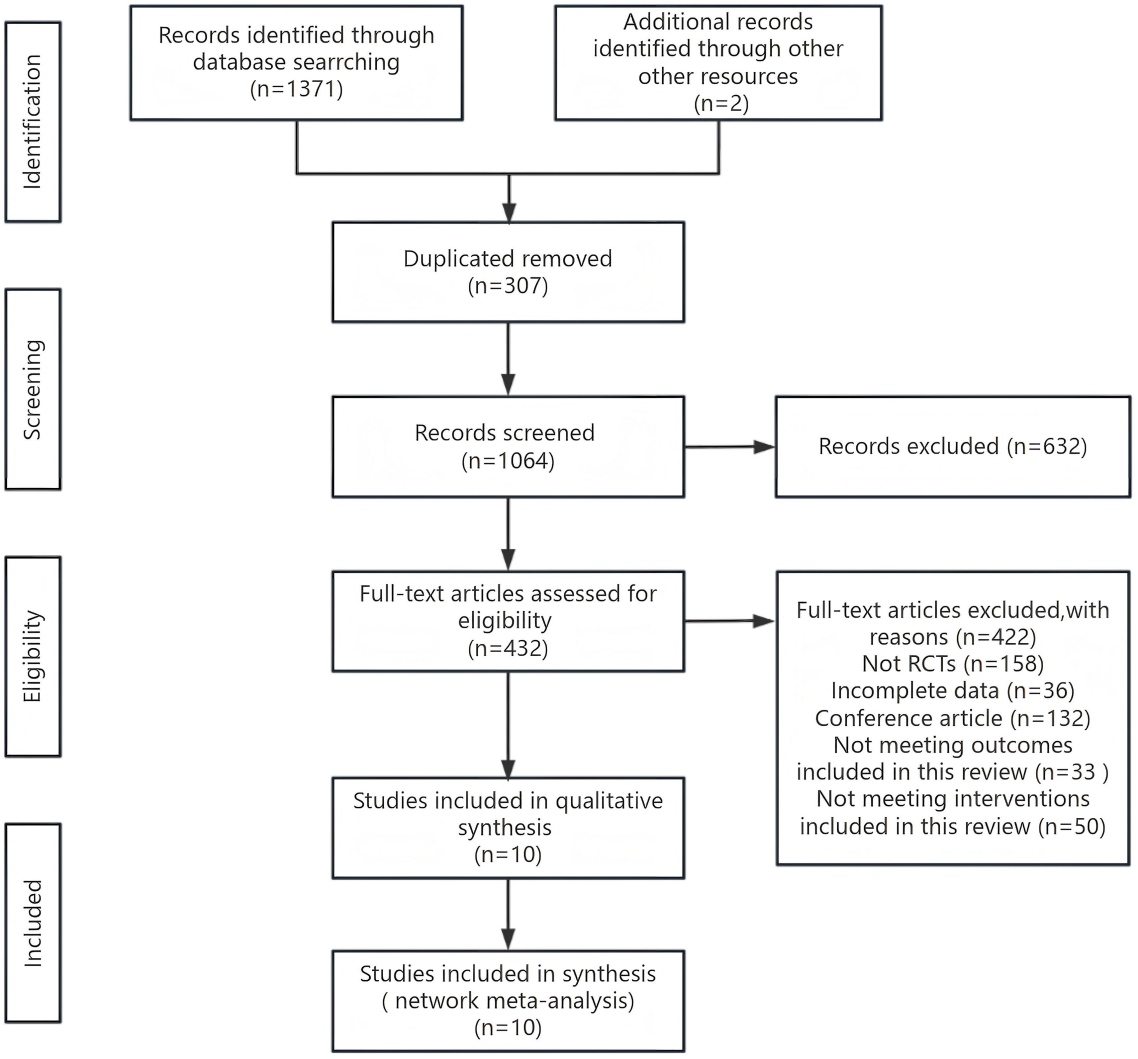
Flow diagram of literature screening.

A total of 1,371 studies were retrieved from four electronic databases, complemented by two studies identified through manual identify. The remaining 1,064 studies were screened according to titles and abstracts after duplicate removal, and 632 studies were subsequently excluded. The remaining 432 studies were thoroughly reviewed, resulting in the exclusion of 422 studies (including incomplete data, reviews, notes, letters, non-RCTs, conference abstracts, and did not come up to the inclusion criteria in this study). Ultimately, 10 studies were incorporated into this meta-analysis (Figure 1).

### 3.2 Quality assessment

**Figure 2:**
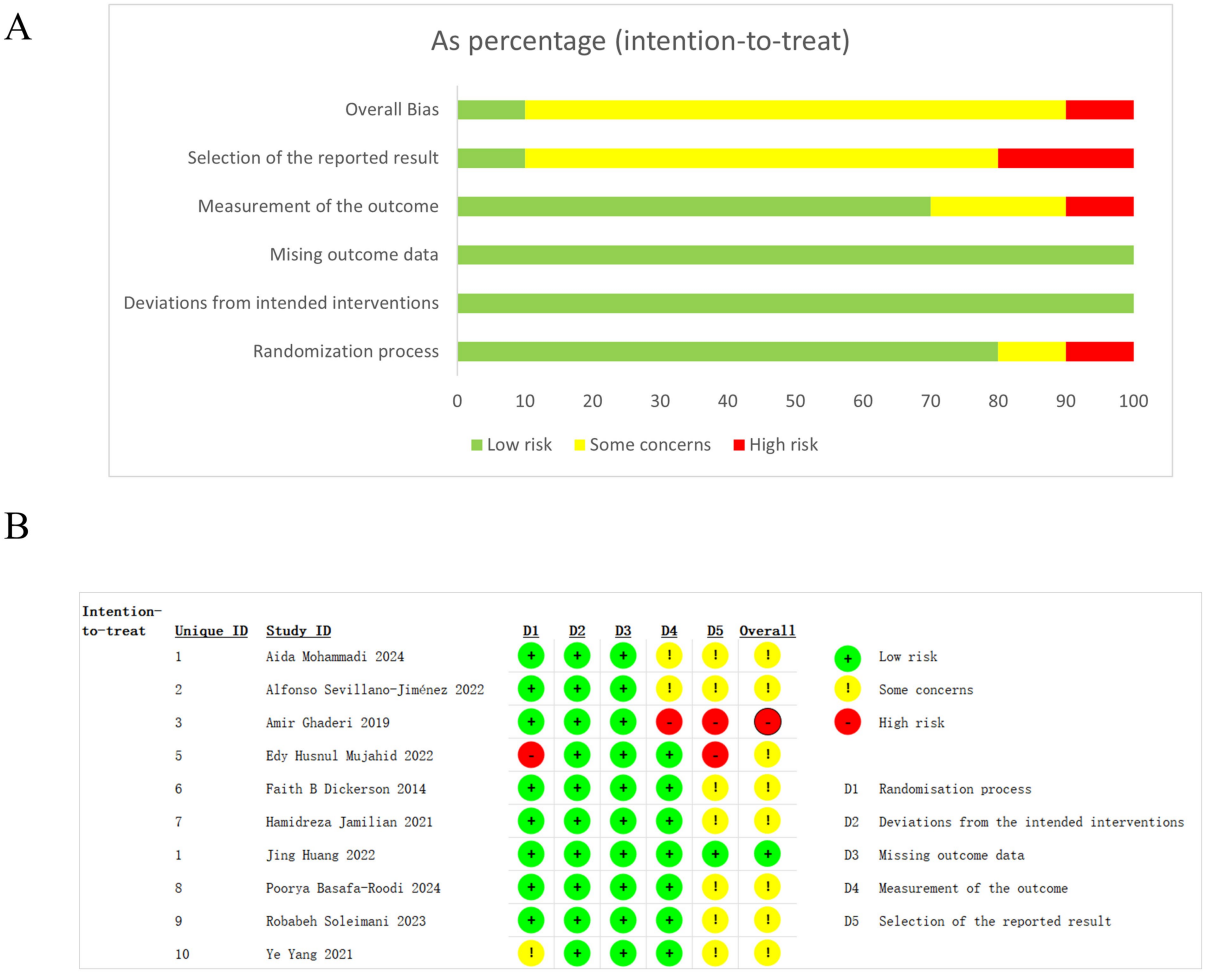
Risk of bias graph **(A)** and summary **(B)** of the included RCTs.

The Cochrane ROB 2.0 assessment tool was employed to evaluate all studies included in this meta-analysis. On the basis of evaluation results, the potential bias of each study was classified as low, high, or unclear. During the randomization process, only two studies (accounting for 20%) were classified as having low risk of bias, and eight as some concern (Figure 2). In studies above, nine achieved simultaneous blinding of both subjects and assessors. Among all included studies, seven included studies investigated probiotic supplementation’s regulatory effects on clinical symptoms, immune function, metabolic indices, and other factors in SCH patients. One study specifically examined synbiotic supplement’s impact on metabolic syndrome in this population, while another assessed auxiliary probiotic dietary regulation on cardiometabolic status in patients with SCH spectrum disorder.

### 3.3 Characteristics of studies

In this study, 10 RCTs comprising 585 SCH-diagnosed patients were analyzed in this investigation (Ghaderi et al., 2019; Yang et al., 2021; Mujahid et al., 2022; Dickerson et al., 2014; Basafa-Roodi et al., 2024; Mohammadi et al., 2024; Soleimani et al., 2023; Huang et al., 2022; Jamilian and Ghaderi, 2021; Sevillano-Jiménez et al., 2022). The intervention treatment ranged from 6 to 14 weeks. The target population consisted of individuals with SCH. All participants were undergoing antipsychotic drug treatment. The probiotics used in these studies were primarily multi-strain formulations, administered in drug forms such as capsules or tablets. The specific strains included Lactobacillus species (e.g., *Lactobacillus acidophilus*, *Lactobacillus reuteri*, *Lactobacillus fermentum*), Bifidobacterium species (e.g., *Bifidobacterium bifidum*, *Bifidobacterium lactis*, *Bifidobacterium longiformis*), and Enterococcus species. The studies were geographically diverse, with five studies from East Asia, two from West Asia, one from Southeast Asia, one from Europe, and one from America, Basic study information is presented in Supplementary Table S5.

### 3.4 Meta-analysis results

#### 3.4.1 Effects on psychiatric symptoms

**Figure 3:**
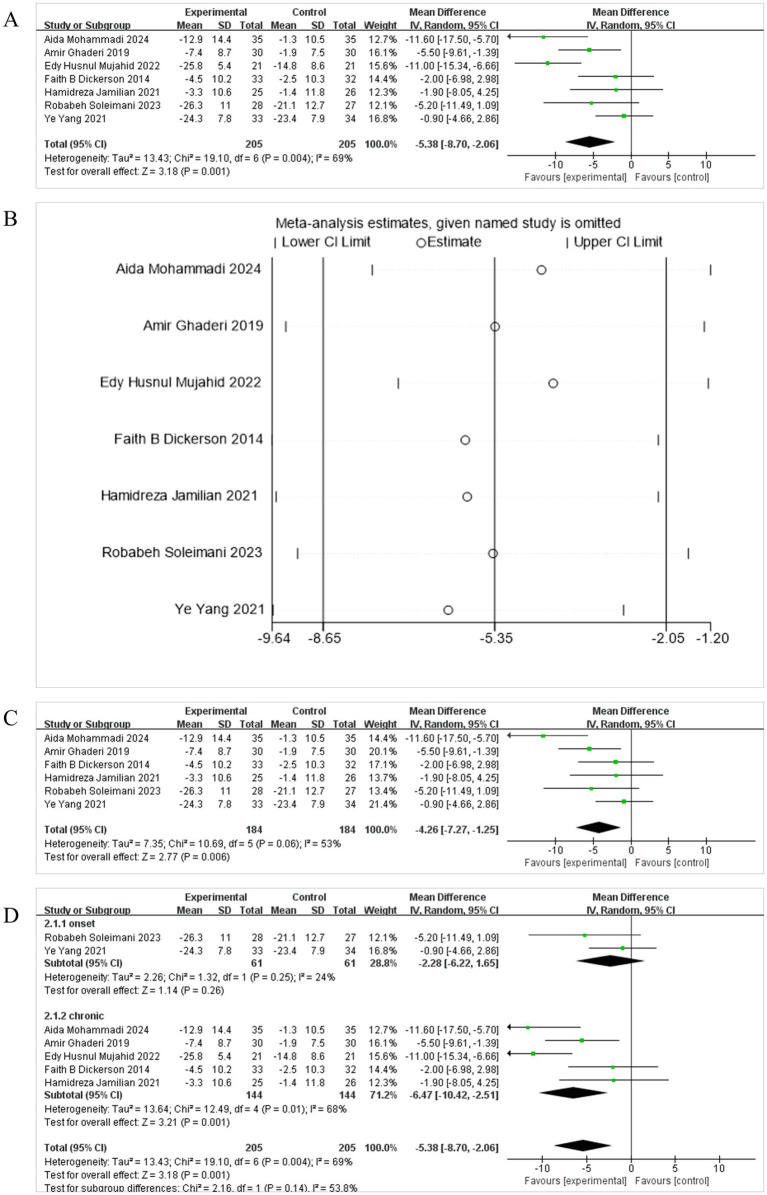
Effect size estimation **(A)**, sensitivity **(B)**, Leave-one-out size estimation **(C)**, and subgroup **(D)** analysis of PANSS.

PANSS assessment revealed a statistically significant difference in psychiatric symptoms between the intervention and placebo groups. Seven studies (Ghaderi et al., 2019; Yang et al., 2021; Mujahid et al., 2022; Dickerson et al., 2014; Mohammadi et al., 2024; Soleimani et al., 2023; Jamilian and Ghaderi, 2021) were included in the analysis related to the total PANSS score, all calculated through the positive and negative symptom scales, so MD was selected. The combined effect was statistically significant [MD = −5.38, 95% CI (−8.70, −2.06)] (Figure 3A), with the diamond plot located to the left of the null line, favoring the probiotics group and not crossing the dashed line. This indicates that, compared with the control group, probiotics administration significantly alleviated clinical symptoms in patients with SCH. There was significant heterogeneity between the studies (*I*^2^ = 69%, *p* = 0.001), so a random-effects model (REM) was employed for the meta-analysis. Sensitivity analysis (Figure 3B) showed that the exclusion of the study by Mujahid et al. (2022) notably influenced the pooled estimate. After its removal, the overall effect size became more pronounced, changing from [MD = −5.38, 95% CI (−8.70, −2.06)] to [MD = −4.26, 95% CI (−7.27, −1.25)], and heterogeneity decreased from *I*^2^ = 69% to *I*^2^ = 53% (Figure 3C). These quantitative changes suggest that this study contributed substantially to the observed heterogeneity, likely due to differences in intervention duration, frequency, and sample size.

Subgroup analysis of PANSS revealed a stage-dependent effect of probiotic supplementation on symptom severity in schizophrenia patients. In chronic patients, probiotics significantly reduced PANSS total scores compared to placebo [MD = −6.47, 95% CI (−10.42, −2.51), *p* = 0.001], with moderate heterogeneity (*I*^2^ = 68%). In contrast, no significant effect was observed in first-onset patients [MD = −2.28, 95% CI (−6.22, 1.65), *p* = 0.26], and heterogeneity was low (*I*^2^ = 24%) (Figure 3D). These findings suggest that probiotics may confer greater clinical benefits in chronic stages of schizophrenia, possibly reflecting differences in gut microbiota composition or immune-inflammatory profiles across disease stages.

**Figure 4:**
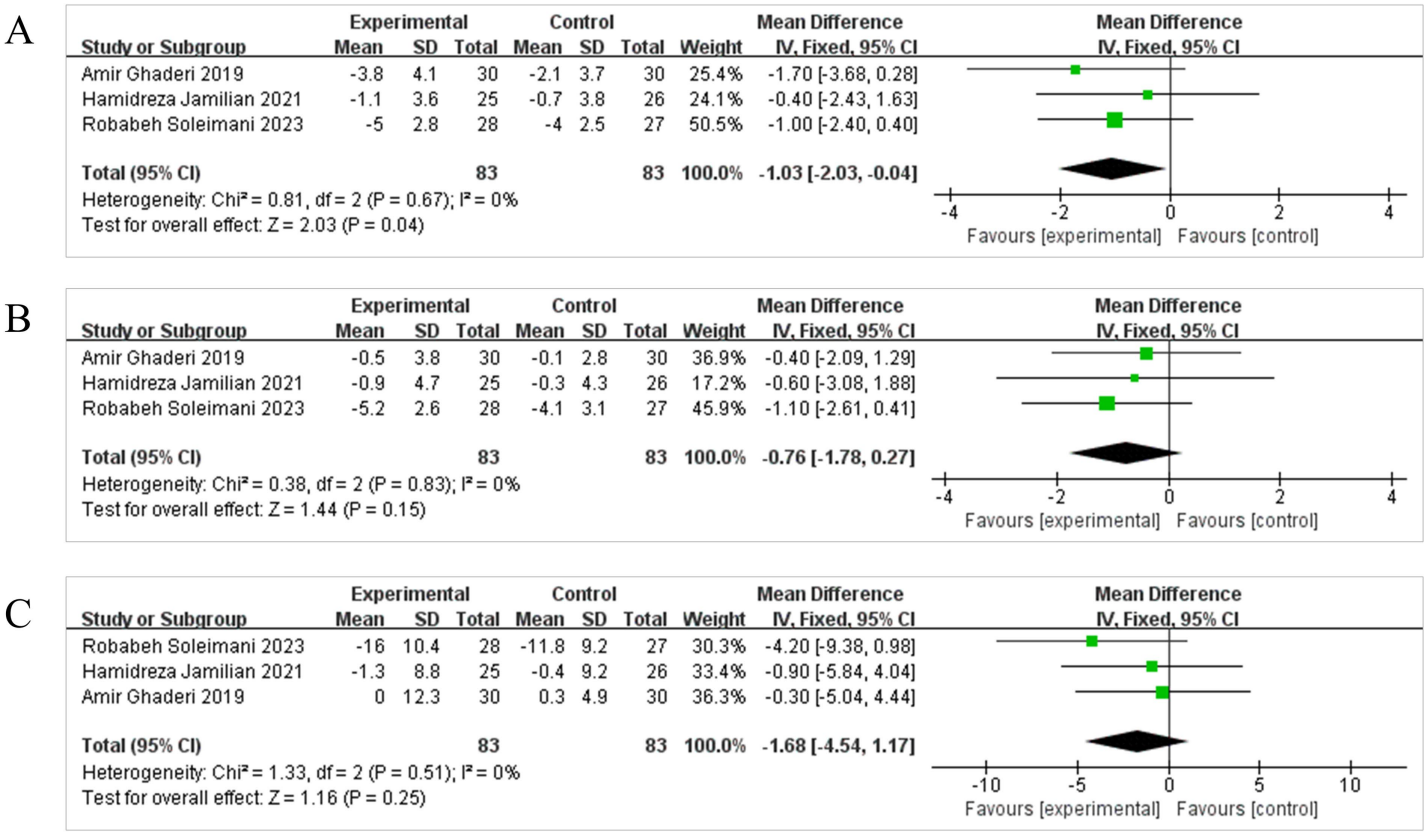
Effect size estimation of PANSS Negative **(A)**, PANSS Positive **(B)**, BPRS **(C)**.

For negative symptoms, as assessed by the PANSS (Figure 4A), the intervention demonstrated statistically superior outcomes relative to placebo. Three studies were incorporated in the analysis of the total PANSS score, and due to negligible heterogeneity (*I*^2^ = 0%, *p* = 0.67), a FEM was applied to the meta-analysis. The combined effect was statistically significant [MD = −1.03, 95% CI (−2.03, −0.04)], and the diamond plot was located to the left of the null line again, favoring the probiotic group, and not intersecting the dashed line. This suggests that, compared to the control group, probiotics administration significantly alleviated negative symptoms in patients with SCH.

Additionally, the absence of statistically significant differences in positive symptoms persisted across both intervention and control groups, as assessed by the PANSS [MD = −0.76, 95% CI (−1.78, 0.27)] (Figure 4B) and the total score of BPRS [MD = −1.68, 95% CI (−4.54, 1.17)] (Figure 4C). Detailed information is presented in the accompanying figure.

#### 3.4.2 Effects on metabolic indicators

##### 3.4.2.1 Blood glucose related indicators

###### 3.4.2.1.1 Effect on FBS levels

**Figure 5:**
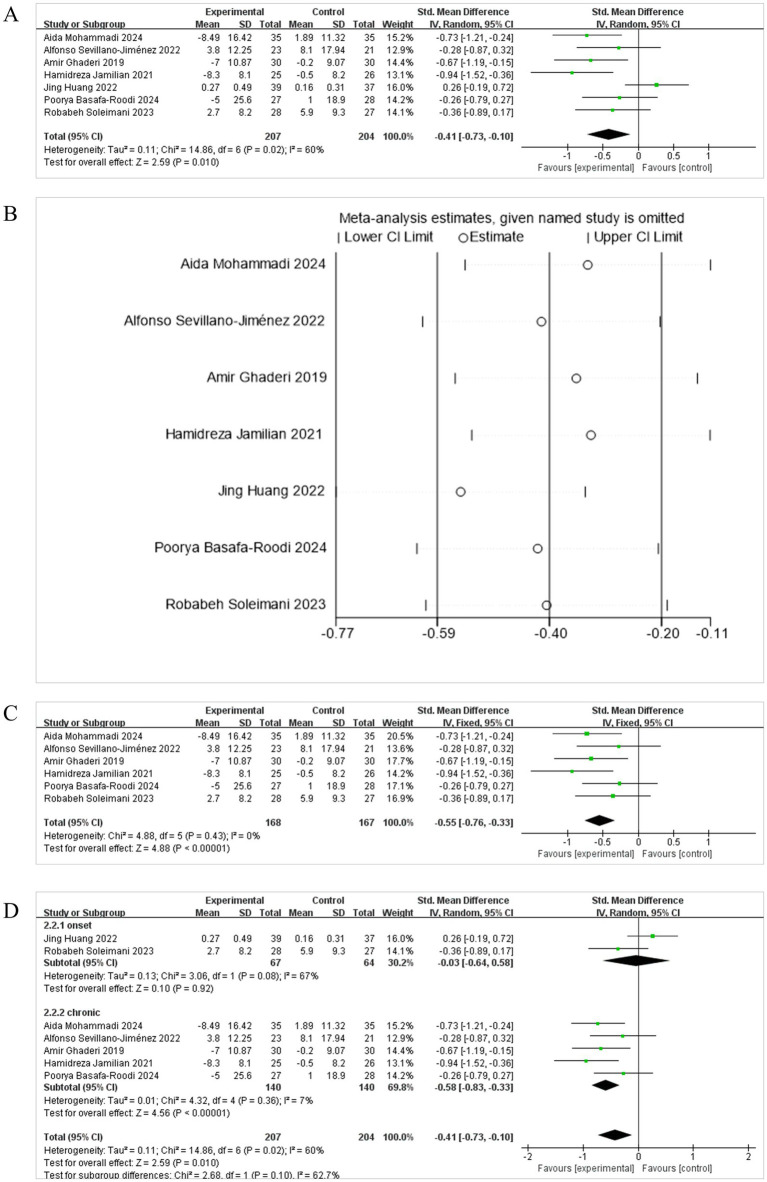
Effect size estimation **(A)**, sensitivity **(B)**, Leave-one-out size estimation **(C)**, and subgroup **(D)** analysis of FBS.

To evaluate the effect of probiotic supplements, synbiotic supplements, and auxiliary probiotic dietary interventions on glucose and lipid metabolism in patients with SCH, using FBS as the outcome indicator, seven studies (Ghaderi et al., 2019; Basafa-Roodi et al., 2024; Mohammadi et al., 2024; Soleimani et al., 2023; Huang et al., 2022; Jamilian and Ghaderi, 2021; Sevillano-Jiménez et al., 2022) met the inclusion criteria. Because of the significant heterogeneity (*I*^2^ = 60%, *p* = 0.010), a REM was used to the meta-analysis. Results indicated a total cohort of 468 participants with a statistically significant combined effect [SMD = −0.41, 95% CI (−0.73, −0.10)] (Figure 5A). The diamond symbol (representing the combined effect) lies to the left of and does not intersect with the line of no effect, suggesting that, compared to placebo or routine diet, adjuvant treatment with auxiliary probiotic supplements, synbiotic supplements, or probiotic dietary interventions was beneficial for reducing FBS levels in patients with SCH. It shows that the sensitivity analysis indicated a significant deviation of the analysis line from that of Huang et al. (2022) beyond the numerical range (Figure 5B), signifying its considerable impact on the stability of the meta-analysis results. After excluding Huang’s study, the overall effect size became more pronounced, changing to [SMD = −0.42, 95% CI (−0.74, −0.10)], and heterogeneity decreased from *I*^2^ = 60% to *I*^2^ = 0% (Figure 5C). These quantitative changes suggest that Huang’s study is the main source of heterogeneity. After excluding it, not only was heterogeneity eliminated, but the effect estimation also became more stable. Although it had no impact on the direction of the effect, it slightly enhanced the overall effect strength.

Besides, subgroup analysis by disease stage showed a significant reduction in FBS in chronic schizophrenia patients [SMD = −0.58, 95% CI (−0.83, −0.33), *p* < 0.00001], with low heterogeneity (*I*^2^ = 7%) (Figure 5D). In contrast, no significant effect was observed in first-onset patients [SMD = −0.03, 95% CI (−0.64, 0.58), *p* = 0.92], and heterogeneity was high (*I*^2^ = 67%). These findings suggest that the glycemic benefits of probiotic supplementation may be stage-dependent, with greater efficacy in chronic populations.

###### 3.4.2.1.2 Effect on INS levels

**Figure 6:**
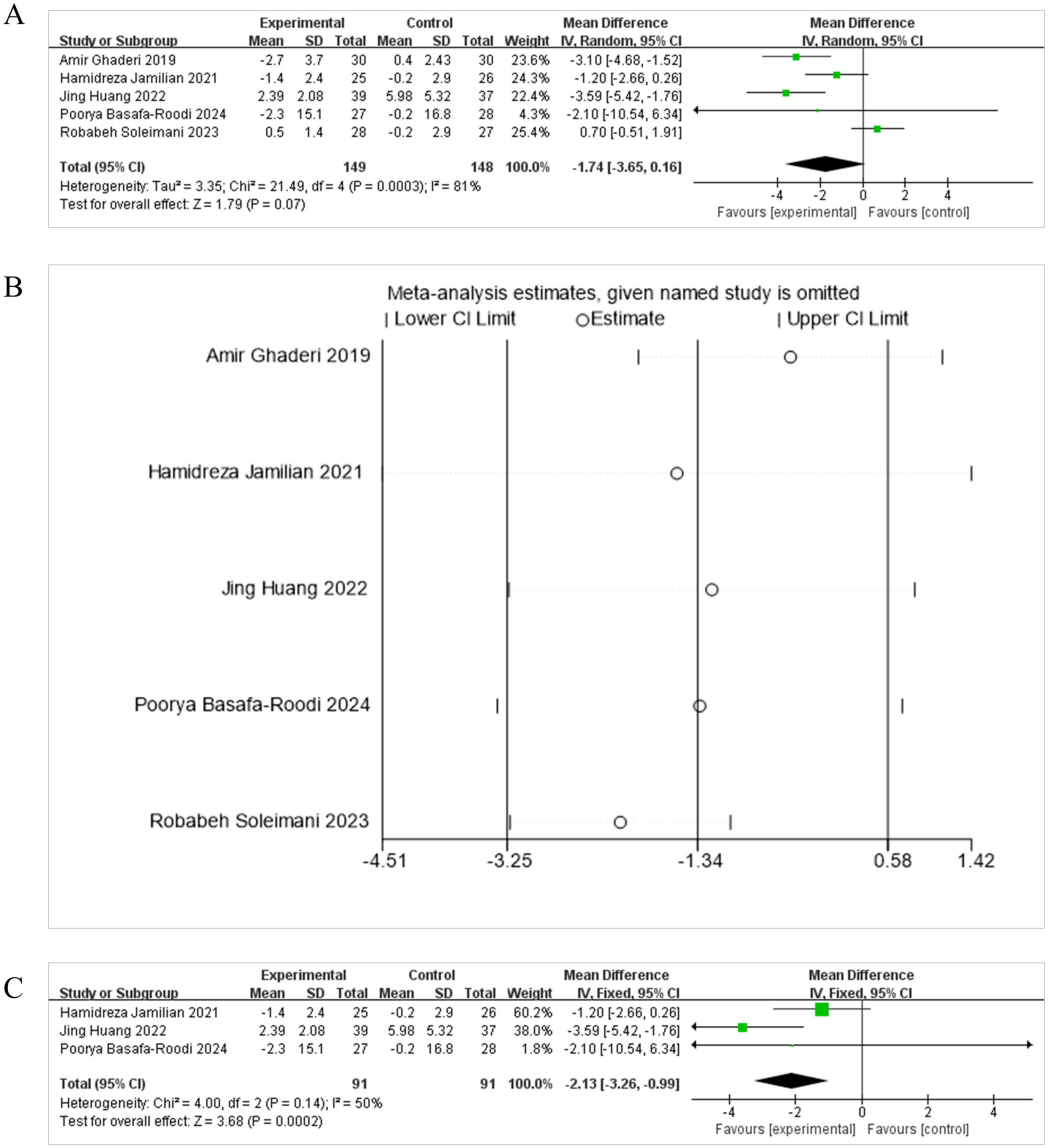
Effect size estimation **(A)**, sensitivity **(B)**, and Leave-one-out size estimation **(C)** analysis of INS.

When INS levels were selected as the outcome indicator, five studies (Ghaderi et al., 2019; Basafa-Roodi et al., 2024; Soleimani et al., 2023; Huang et al., 2022; Jamilian and Ghaderi, 2021) were brought into the meta-analysis. Due to significant heterogeneity (*I*^2^ = 81%, *p* = 0.07), a REM was utilized for the meta-analysis. The results unmasked the combined effect was statistically significant [MD = −1.74, 95% CI (−3.65, 0.16)] (Figure 6A). The diamond intersected with the line of no effect. This suggests that compared to the control group, the adjunctive probiotic intervention was not beneficial for reducing INS levels.

The sensitivity analysis showed the analysis line of Ghaderi et al. (2019) and Soleimani et al. (2023) significantly deviated from the numerical range, indicating its considerable impact on the stability of the meta-analysis results (Figure 6B). After excluding the two outlying studies the remaining three trials (Jamilian and Ghaderi, 2021; Huang et al., 2022; Basafa-Roodi et al., 2024) exhibited moderate and non-significant heterogeneity (*I*^2^ = 50%, *p* = 0.14). A fixed-effect model yielded a significant pooled [MD = −2.13, 95% CI (−3.26, −0.99), *p* = 0.0002], with the diamond entirely on the left of the no-effect line (Figure 6C). Taken together, the body of evidence is sensitive to the inclusion of highly weighted outliers. Once these trials are removed, adjunctive probiotic or synbiotic therapy appears to confer a clinically relevant reduction in fasting insulin levels. Nevertheless, the fragility of the result underscores the need for larger, methodologically homogeneous studies before firm therapeutic claims can be made.

##### 3.4.2.2 Lipid-related indicators

###### 3.4.2.2.1 Effect on TG levels

**Figure 7:**
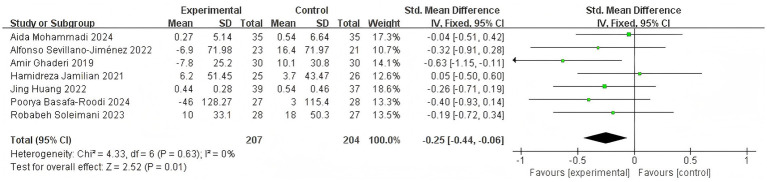
Effect size estimation of TG.

When TG levels were designated as the outcome measure, seven studies qualified for inclusion (Ghaderi et al., 2019; Basafa-Roodi et al., 2024; Mohammadi et al., 2024; Soleimani et al., 2023; Huang et al., 2022; Jamilian and Ghaderi, 2021; Sevillano-Jiménez et al., 2022). Due to negligible heterogeneity, a FEM was employed for the meta-analysis (*I*^2^ = 0%, *p* = 0.01). Analysis revealed a statistically significant combined effect [SMD = −0.25, 95% CI (−0.44, −0.06)] (Figure 7). The diamond was positioned to the left and did not intersect with the boundary of the ineffective area, indicating that probiotic or synbiotic adjuvant intervention was more effective than the control group in reducing TG levels.

###### 3.4.2.2.2 Effect on total TC levels

**Figure 8:**
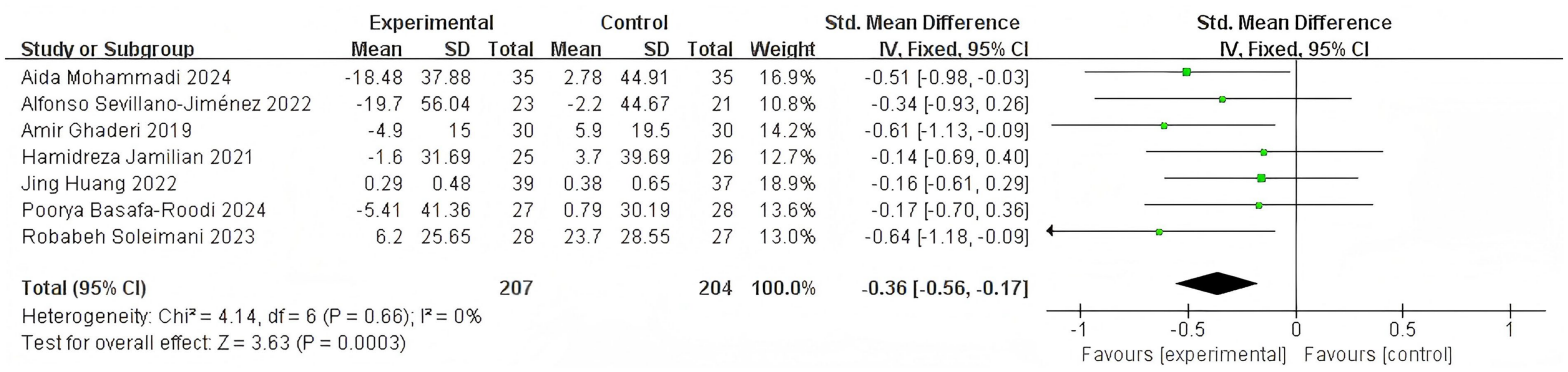
Effect size estimation of TC.

When total cholesterol (TC) levels were designated as the outcome measure, seven studies qualified for inclusion (Ghaderi et al., 2019; Basafa-Roodi et al., 2024; Mohammadi et al., 2024; Soleimani et al., 2023; Huang et al., 2022; Jamilian and Ghaderi, 2021; Sevillano-Jiménez et al., 2022). Because of the negligible heterogeneity, a FEM was employed for the meta-analysis (*I*^2^ = 0%, *p* = 0.0003). The results demonstrated that the combined effect was statistically significant [SMD = −0.36, 95% CI (−0.56, −0.17)] (Figure 8). The diamond was positioned to the left and did not intersect with the line of no effect, indicating that probiotic or synbiotic adjuvant supplement was beneficial to TC levels compared to the control group.

###### 3.4.2.2.3 Effect on HDL-cholesterol levels

**Figure 9:**
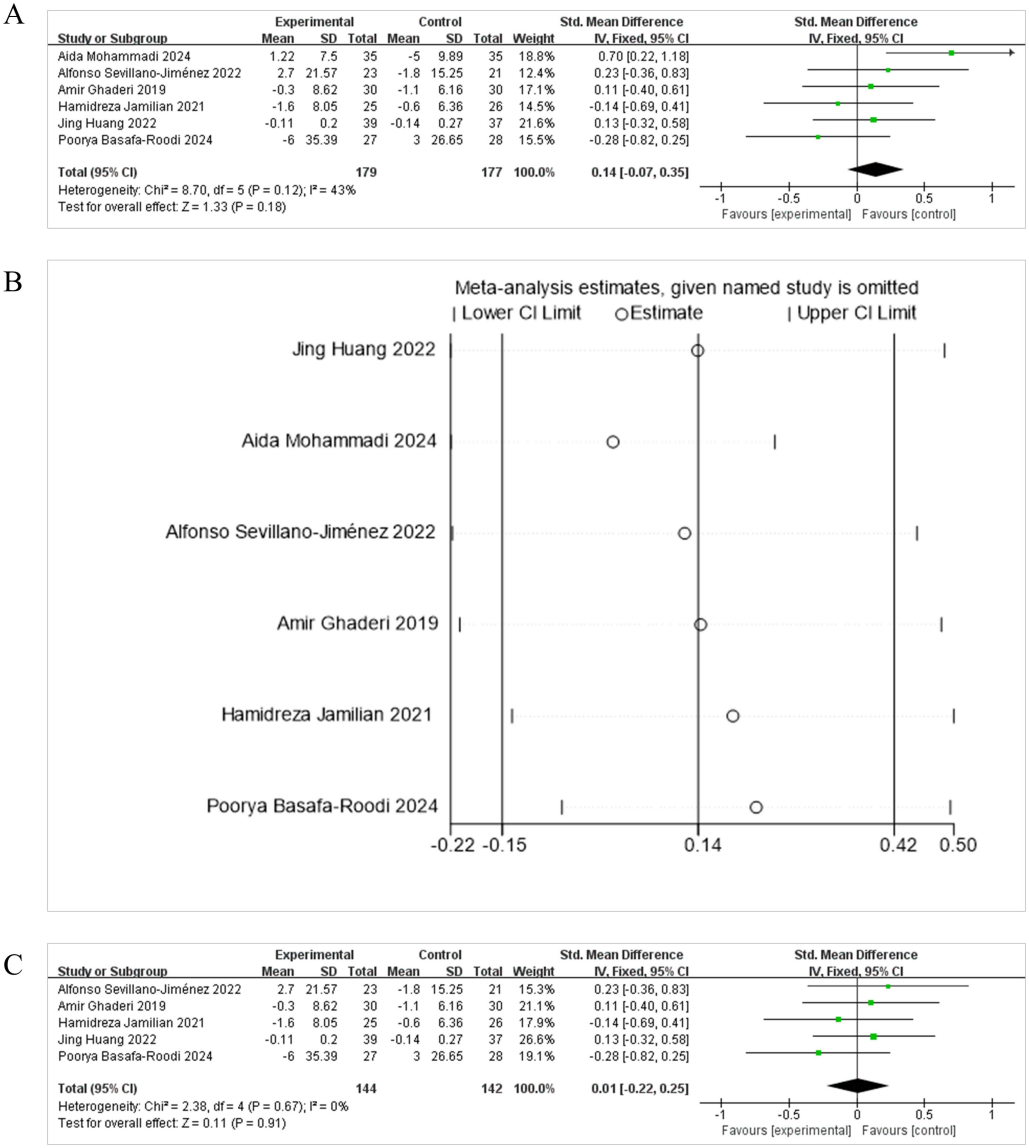
Effect size estimation **(A)**, sensitivity **(B)**, and Leave-one-out size estimation **(C)** analysis of HDL-cholesterol.

When HDL-cholesterol levels were selected as the outcome indicator, six studies were included (Ghaderi et al., 2019; Basafa-Roodi et al., 2024; Mohammadi et al., 2024; Huang et al., 2022; Jamilian and Ghaderi, 2021; Sevillano-Jiménez et al., 2022). As the heterogeneity was not significant, a FEM was employed for the meta-analysis (*I*^2^ = 43%, *p* = 0.18). The results demonstrated that the combined effect was not statistically significant [SMD = 0.14, 95% CI (−0.07, 0.35)] (Figure 9A). The diamond was positioned to the right and intersected with the line of no effect, indicating that probiotic, prebiotic or synbiotic adjuvant therapy was not more effective than placebo therapy in reducing HDL-cholesterol levels, as evidenced by the diamond overlapping with the null line.

The sensitivity analysis showed the analysis line of Mohammadi et al. (2024) significantly deviated from the numerical range, indicating its considerable impact on the stability of the meta-analysis results (Figure 9B). Leave-one-out diagnostics identified the trial by Mohammadi et al. (2024) as an influential outlier whose exclusion abolished heterogeneity (*I*^2^ = 0%) and shifted the pooled [SMD = 0.01, 95% CI (−0.22, 0.25), *p* = 0.91], centring almost exactly on zero (Figure 9C). Thus, the modest positive trend observed in the full dataset was driven by a single study; after its removal there is no credible evidence that gut-directed therapy raises HDL-cholesterol in patients with schizophrenia. Larger, methodologically consistent trials are required before any cardiometabolic advantage can be claimed.

###### 3.4.2.2.4 Effect on LDL-cholesterol levels

**Figure 10:**
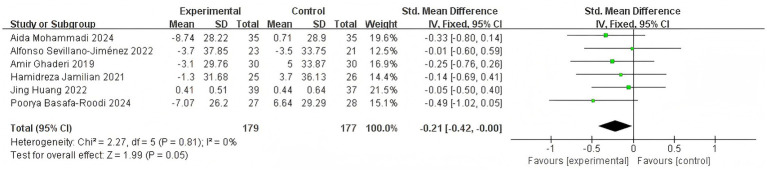
Effect size estimation of LDL-cholesterol.

When LDL-cholesterol levels were selected as the outcome indicator, six studies were included (Ghaderi et al., 2019; Basafa-Roodi et al., 2024; Mohammadi et al., 2024; Huang et al., 2022; Jamilian and Ghaderi, 2021; Sevillano-Jiménez et al., 2022). Due to negligible heterogeneity, a FEM was employed for the meta-analysis (*I*^2^ = 0%, *p* = 0.05). The results demonstrated that the combined effect was not statistically significant [MD = −0.21, 95% CI (−0.42, 0.00)] (Figure 10). The diamond intersected with the null line, indicating that probiotic, prebiotic or synbiotic adjuvant therapy was not more effective than placebo therapy in reducing LDL-cholesterol levels, as evidenced by the diamond overlapping with the null line.

##### 3.4.2.3 Effects on insulin-related indicators

###### 3.4.2.3.1 Effect on HOMA-IR levels

**Figure 11:**
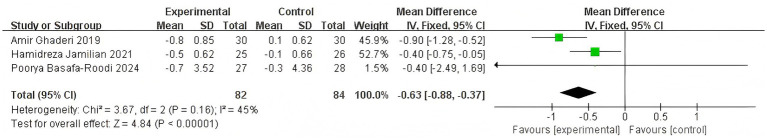
Effect size estimation of HOMA-IR.

When HOMA-IR levels were selected as the outcome indicator, three studies were included (Ghaderi et al., 2019; Basafa-Roodi et al., 2024; Jamilian and Ghaderi, 2021), and the HOMA-IR were calculated according to the same suggested formulas (Pisprasert et al., 2013), so MD was adopted. Because of significant heterogeneity (*I*^2^ = 45%, *p* < 0.00001), a FEM was employed for the meta-analysis. The results demonstrated that the combined effect was statistically significant [MD = −0.63, 95% CI (−0.88, −0.37)] (Figure 11). The diamond was located to the left and did not intersect with the null line, indicating that probiotic or prebiotic adjuvant therapy was beneficial to HOMA-IR levels and had a positive effect on reducing metabolic abnormalities and cardiovascular disease risk in patients.

###### 3.4.2.3.2 Effect on QUICKI levels

**Figure 12:**
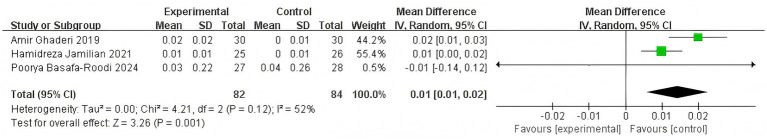
Effect size estimation of QUICKI.

When QUICKI levels were selected as the outcome indicator, three studies were included (Ghaderi et al., 2019; Basafa-Roodi et al., 2024; Jamilian and Ghaderi, 2021), and the QUICK were calculated according to the same suggested formulas (Pisprasert et al., 2013), so MD was adopted. As the heterogeneity was significant (*I*^2^ = 52%, *p* = 0.001), a REM was employed for the meta-analysis. The results demonstrated that the combined effect was statistically significant [MD = 0.01, 95% CI (0.01, 0.02)] (Figure 12). Probiotic or synbiotic adjuvant therapy helpfully improved QUICKI levels, as evidenced by the diamond’s non-intersection with the null line.

#### 3.4.3 Effects on physiological indicators

##### 3.4.3.1 Effect on BW levels

**Figure 13:**
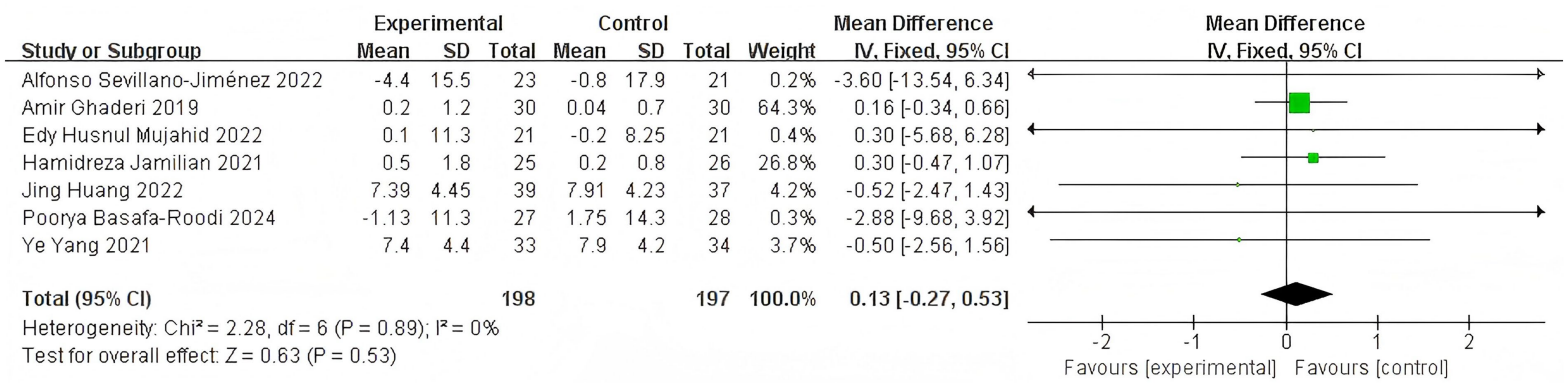
Effect size estimation of BW.

When BW levels were selected as the outcome indicator, seven studies were incorporated into the meta-analysis (Ghaderi et al., 2019; Yang et al., 2021; Mujahid et al., 2022; Basafa-Roodi et al., 2024; Huang et al., 2022; Jamilian and Ghaderi, 2021; Sevillano-Jiménez et al., 2022). Given the negligible heterogeneity (*I*^2^ = 0%, *p* = 0.53), a FEM was employed for the meta-analysis (Figure 13). The results revealed that the combined effect was not statistically significant [MD = 0.13, 95% CI (−0.27, 0.53)]. The diamond crossed the line of no effect indicates that probiotic adjuvant therapy showed no statistically significant advantage over placebo therapy in reducing BW levels, as demonstrated by the diamond in the figure below.

##### 3.4.3.2 Effect on BMI levels

**Figure 14:**
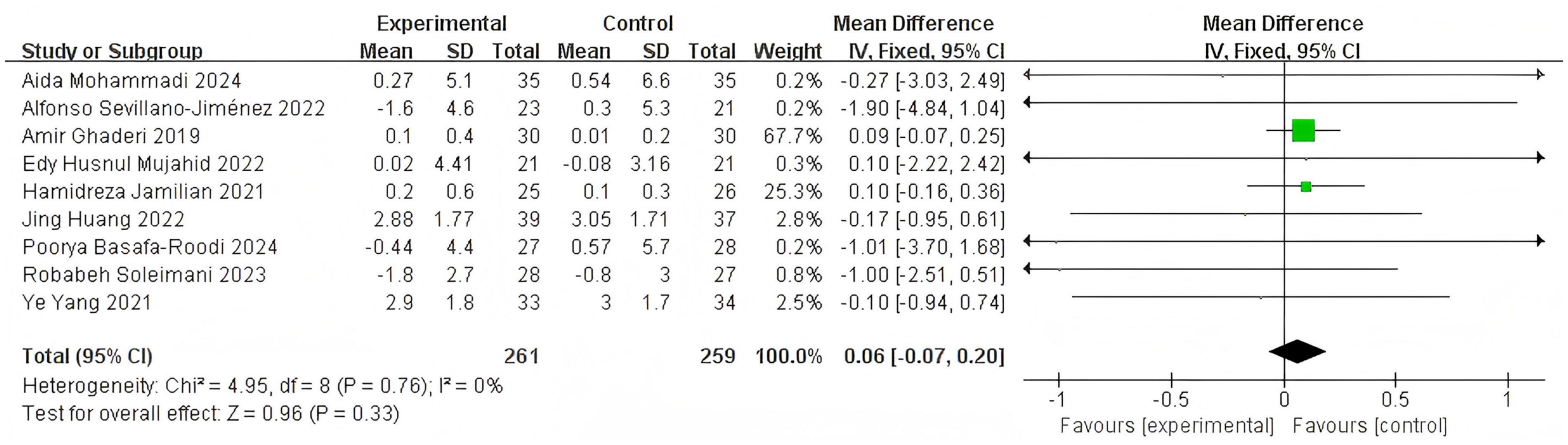
Effect size estimation of BMI.

When BMI was selected as the outcome measure, nine studies were incorporated into the meta-analysis (Ghaderi et al., 2019; Yang et al., 2021; Mujahid et al., 2022; Basafa-Roodi et al., 2024; Mohammadi et al., 2024; Soleimani et al., 2023; Huang et al., 2022; Jamilian and Ghaderi, 2021; Sevillano-Jiménez et al., 2022). Given the negligible heterogeneity observed (*I*^2^ = 0%, *p* = 0.33), a FEM was employed for the meta-analysis (Figure 14). The results revealed that the combined effect was not statistically significant [MD = 0.06 95% CI (−0.07, 0.20)]. The diamond crossed the null line signifies that probiotic adjuvant therapy was not significantly more effective than placebo in reducing BMI levels, a finding visually confirmed by the diamond plot below.

### 3.5 Qualitative description of probiotics and non-probiotics studies

In fact, the intervention measures for the gut microbiota on patients with schizophrenia include probiotics/synbiotics/diet (even fecal microbial transplantation has also been mentioned), but the studies included in this research mainly focused on probiotics (8 out of 10), while the evidence for other non-probiotic intervention measures is relatively scarce.

In non-probiotic interventions, the studies by Sevillano-Jiménez et al. (2022) and Basafa-Roodi et al. (2024) provide valuable insights into the metabolic effects of dietary education and synbiotic supplementation in individuals with schizophrenia. Across two placebo-controlled RCTs that evaluated symbiotic or probiotic interventions in schizophrenia-spectrum patients with pre-existing metabolic disturbances, the evidence points to a consistent, albeit modest, improvement in anthropometric and glycaemic indices. Sevillano-Jiménez et al. (2022) (*n* = 44, 6-month high-symbiotic diet) observed a 27.4% relative reduction in the cumulative prevalence of metabolic-syndrome components (*p* > 0.05 for between-group difference) driven by decreasing waist circumference (−3.7 cm), BMI (−1.6 kg/m^2^) and diastolic BP (−4.8 mmHg) in the intervention arm, with no parallel change in fasting glucose or lipids. Basafa-Roodi et al. (2024) (*n* = 55, 8 week synbiotic capsules) reported a statistically significant advantage over placebo for waist circumference (−2.7 cm vs. +3.0 cm; *p* < 0.001), HbA1c (−0.26% vs. + 0.20%; *p* = 0.005) and attenuation of antipsychotic-related BMI gain (−0.37 kg/m^2^ vs. + 0.61 kg/m^2^; *p* = 0.01), while LDL-cholesterol and triglycerides fell only within the synbiotic group. Taken together, these trials yield that targeted probiotics dietary and symbiotic supplementation can beneficially modify central adiposity and glycaemic control in this high-risk population; however, effects on hard cardiometabolic end-points remain untested and the absence of microbiome sequencing precludes mechanistic inference.

**Figure 15:**
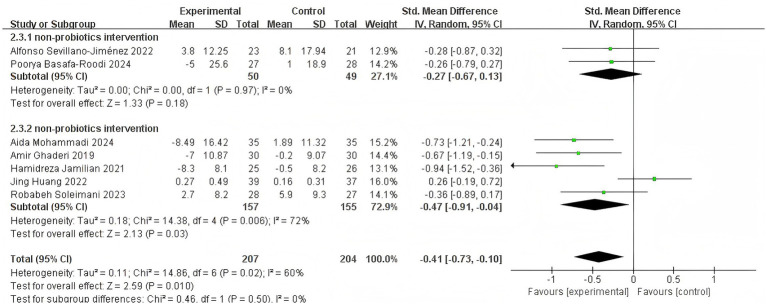
Effect size estimation of the probiotic group and the non-probiotic group in FBS.

Taking FBS as an example (Figure 15), non-probiotic intervention (two studies, *n* = 99) deliver almost null glycaemic change: pooled SMD = −0.27 (95% CI (–0.67, 0.13), *p* = 0.18, *I*^2^ = 0%). Sevillano-Jiménez et al. (2022) (dietary education) and Basafa-Roodi et al. (2024) (capsular synbiotic without live bacteria) report mean FBS changes of +3.8 mg dL^−1^ and −5 mg dL^−1^, respectively—both within the biological variation of fasting glucose. While probiotics intervention (five RCTs, *n* = 312) shift FBS downward: pooled SMD = −0.47 (95% CI (–0.91, −0.04), *p* = 0.03, *I*^2^ = 72%). The individual mean reductions range from −8.5 mg dL^−1^ (Mohammadi et al., 2024) to −7 mg dL^−1^ (Ghaderi et al., 2019) and −8.3 mg dL^−1^ (Jamilian and Ghaderi, 2021), whereas the small-scale Huang et al. (2022) trial shows a trivial +0.3 mg dL^−1^ change, explaining the observed heterogeneity.

Overall, the random-effects meta-analysis across all seven trials yields a significant combined SMD = −0.41 (95% CI (–0.73, 0.10), *p* = 0.010, *I*2 = 60%) favoring intervention; however, 60% of the weight comes from the probiotic cluster. Thus, when quantity and effect are considered together, probiotics interventions appear to drive the observable glucose-lowering signal, whereas non-probiotic dietary or synbiotic capsule approaches contribute neutral evidence. This quantitative separation supports the editorial suggestion to narratively synthesize non-probiotic studies separately rather than merging them indiscriminately with probiotic data.

### 3.6 Publication bias test

Formal statistical testing for publication bias (e.g., using funnel plots or Egger’s test) was not performed for any outcome, as the number of included studies (a maximum of 9 for BMI) was below the conventional threshold of 10 required for reliable interpretation (Sterne et al., 2011). Nevertheless, we acknowledge the potential for publication bias and have addressed it qualitatively. Many included trials are small, and small trials with non-significant results are less likely to be published. Furthermore, industry sponsorship is common in probiotic research, which is a known source of bias potentially favoring the publication of positive outcomes. Although our comprehensive search strategy included gray literature to minimize this risk, the overall results should still be interpreted with caution, as the pooled effect estimates might be inflated due to missing negative studies.

### 3.7 Evidence certainty of GRADE assessment

In this study, GRADEPro3.6 software was applied to grade the evidence quality of 14 outcome indicators (PANSS, negative PANSS, positive PANSS, BPRS, FBS, INS, TG, TC, HDL cholesterol, LDL cholesterol, HOMA-IR, QUICKI, BW, BMI). The findings indicated that only the evidence quality of the Negative PANSS, one of the outcome indicators, was moderate. This moderate-quality evidence enhanced the overall effective rate of gut microbiota intervention in schizophrenia patients and the credibility of satisfaction evidence. However, the evidence quality of the remaining 13 outcome measures was either low or very low (Supplementary Table S4). Even after sensitivity analysis, heterogeneity persisted, which might influence the reliability of the conclusion. Consequently, in future research, researchers should standardize the experimental design and strictly execute the research process.

## 4 Discussion

### 4.1 Summary of evidence

In our study, we implemented a comparative assessment of the efficacy and impact of gut microbiota-targeted interventions in alleviating psychopathological symptoms and improving metabolic parameters in SCH patients. The meta-analysis incorporated 10 clinical trials encompassing three specific adjunctive probiotic therapeutic modalities: probiotic supplementation, synbiotic supplementation, and probiotic dietary modifications, involving a total cohort of 783 SCH patients. Our analysis demonstrated that probiotic intervention groups performed better than the placebo control group in terms of psychopathological symptoms and metabolic indicators. The therapeutic benefits were evidenced by reductions in PANSS scores and significant improvements in multiple metabolic indices, including FBS, INS, TG, TC, HOMA-IR and QUICKI. These findings collectively suggest that gut microbiota modulation represents a clinically viable intervention strategy for concurrent improvement of psychiatric manifestations and metabolic dysregulation in SCH patients. It is important to note that the beneficial effects were not universal across all measured metabolic parameters. Specifically, the intervention did not result in significant changes in BW, BMI, or HDL/LDL levels, suggesting a more targeted rather than a generalized metabolic effect.

Regarding the impact on psychopathological symptoms in patients with SCH, gut microbiota modulation via probiotics demonstrated efficacy in reducing the PANSS scores and improving the negative symptoms. However, it did not significantly impact the BPRS scores or improve the positive symptoms. This dissociation can be interpreted through a gut-brain-axis framework that links microbial ecology to discrete symptom dimensions of schizophrenia (Szeligowski et al., 2020). First, negative and cognitive symptoms have been closely associated with chronic low-grade inflammation, reduced hippocampal neurogenesis, and diminished dopaminergic and glutamatergic neurotransmission—pathological processes modulated by microbial metabolites such as short-chain fatty acids (SCFAs) and tryptophan-derived indoles. Probiotic administration restores beneficial taxa (e.g., Lactobacillus, Bifidobacterium, Saccharomyces) and enriches SCFA-producing communities, thereby elevating levels of butyrate and propionate. These metabolites enhance blood–brain barrier integrity, suppress pro-inflammatory cytokines (e.g., IL-6, TNF-α), and upregulate neurotrophic factors such as BDNF (Jamilian and Ghaderi, 2021; Mohammadi et al., 2024). These mechanisms preferentially target domains such as apathy, anhedonia, and cognitive impairment—captured by the PANSS negative subscale—while exerting limited influence on mesolimbic hyperdopaminergic circuits implicated in hallucinations and delusions. Second, positive symptoms are primarily linked to acute striatal dopamine dysregulation, a pathway less directly influenced by gut microbiota composition. Although cross-sectional metagenomic studies have reported correlations between microbial abundance (e.g., Lactobacillus) and PANSS positive scores (Zhu et al., 2020), interventional trials have not demonstrated consistent clinical benefits, suggesting that probiotic modulation alone is insufficient to counteract entrenched dopaminergic dysfunction, whether drug-naïve or antipsychotic-affected. Third, methodological limitations may contribute to the null findings regarding positive symptoms. The median duration of interventions ranged from 8 to 12 weeks, whereas detectable modulation of positive symptoms may require extended periods (≥6 months) of immune and neuroplastic adaptation (Dickerson et al., 2014; Soleimani et al., 2023). Furthermore, the aggregated sample sizes (median *n* = 60) were underpowered to detect small effect sizes (Cohen’s *d* < 0.30) on the BPRS, which also exhibits significant heterogeneity across sites due to variations in rater training and cultural-linguistic factors. In summary, current evidence suggests that probiotic interventions exert their benefits primarily through anti-inflammatory and neurotrophic mechanisms aligned with the pathophysiology of negative and cognitive symptoms, rather than through direct modulation of dopaminergic pathways involved in positive symptoms. Future multi-centre trials featuring longer follow-up, larger samples, and combined metagenomic and neuroimaging biomarkers are needed to validate these pathway-specific effects.

Meanwhile, the available evidence did not demonstrate significant effects of probiotics on certain metabolic parameters (e.g., HDL, LDL), the significant improvement observed in psychiatric symptoms can be plausibly explained through the gut-brain axis mechanisms. The gut-brain axis is a complex, bidirectional communication network linking the enteric nervous system to the central nervous system. Probiotics may exert their psychotropic effects through several key pathways: Firstly, they can produce and modulate a range of neuroactive metabolites, including short-chain fatty acids (SCFAs), gamma-aminobutyric acid (GABA), and serotonin precursors, which can systemically influence brain function and behavior (Silva et al., 2020; Dezfouli et al., 2024). Secondly, by promoting intestinal barrier integrity and reducing systemic inflammation—a known contributor to neuroinflammation and depression—probiotics may indirectly ameliorate psychiatric symptoms (Foster et al., 2017). Lastly, afferent vagal nerve signaling has been shown to be a critical route through which gut microbes communicate with the brain, influencing emotional behavior (Bravo et al., 2011). Therefore, the positive findings for psychiatric outcomes in this meta-analysis are strongly supported by a growing body of evidence from preclinical and clinical studies on the microbiota-gut-brain axis, providing a compelling rationale for the use of probiotics in mental health.

In terms of the effects on metabolic indices of SCH patients, probiotic-based intestinal microbiota intervention is beneficial for improving the following metabolic indices: blood glucose-related indicators (e.g., FBS, INS), lipid-related indicators (e.g., TG, TC), and insulin-related indicators (e.g., HOMA-IR, QUICKI). No significant effects were observed on HDL-cholesterol, LDL-cholesterol and physical measurement indices (e.g., WC, BW and BMI). The lack of effects can be primarily attributed to the following factors.

Firstly, regarding lipid metabolism, severe RCTs conducted by Ghaderi et al. (2019), Basafa-Roodi et al. (2024), and Soleimani et al. (2023) have demonstrated that probiotic supplementation significantly reduces serum total cholesterol and triglycerides. The underlying mechanisms for this effect, as suggested by pre-clinical evidence, may involve several pathways, including the binding of cholesterol to bacterial cell membranes, the inhibition of intestinal cholesterol absorption, and the modulation of bile acid metabolism (Mohammadi et al., 2024). These processes are thought to be mediated through the activation of specific regulatory proteins that facilitate cholesterol assimilation. Furthermore Zhu et al. (2023) and Chen et al. (2024) reported increased levels of short-chain fatty acids (SCFAs) following probiotic intervention. Based on their evidence from experimental models, these microbial-derived metabolites may regulate host lipid metabolism and ameliorate insulin resistance through GPR41 and GPR43 receptor activation pathways.

Secondly, regarding immunomodulatory effects, as reported in two RCTs (Jamilian and Ghaderi, 2021; Ghaderi et al., 2019), probiotic intaking is associated with a significant reduction in systemic inflammatory biomarkers, particularly high-sensitivity C-reactive protein (hs-CRP) (Chen et al., 2023). To explain this anti-inflammatory effect observed in the RCTs, the suppression of the TLR4/MyD88/NF-κB signaling cascade is a hypothesized pathway through which probiotics could exert immunomodulatory effects (Ling et al., 2022). This mechanism, primarily observed in animal and cell culture models, suggests that probiotics may promote gut microbiota homeostasis, enhance the abundance of beneficial bacteria, and strengthen intestinal barrier integrity. A consequent reduction in the leakage of pro-inflammatory mediators from the gut into circulation could explain the improved systemic antioxidant capacity and lower inflammation seen in our clinical results.

Thirdly, regarding anthropometric outcomes, the improvements in lipid metabolism and immune regulation induced by adjunctive probiotics may indirectly contribute to a reduction in waist circumference through systemic metabolic optimization. The fact that there is no significant reduction in weight or body mass index (BMI) despite the metabolic benefits can be accounted for by several factors. Many of the interventions have a relatively short duration, which may not be sufficient to translate metabolic changes into observable weight loss, especially in populations experiencing weight gain induced by antipsychotic drugs. This weight gain process is mainly mediated by hypothalamic appetite dysregulation, metabolic adaptations, and endocrine disruptions. Furthermore, probiotics act through multimodal pathways, including microbial modulation, metabolic signaling, and anti-inflammatory mechanisms, rather than directly targeting adipose tissue accumulation. As a result, their effectiveness in alleviating weight gain may be partial and context-dependent, particularly when used as monotherapy (Jamilian and Ghaderi, 2021). And methodological limitations across studies, such as small sample sizes and variable intervention designs, further complicate the consistent detection of anthropometric effects.

As for the anthropometric indices, probiotic-dominant gut microbiota intervention had no significant impact on BW and BMI. This may be attributed to the complex involvement of multiple metabolic pathways in weight regulation and BMI alterations, as discussed previously, including insulin resistance, inflammatory responses, and microbial metabolites (e.g., SCFAs). Although probiotics exhibit therapeutic potential in ameliorating certain metabolic parameters (e.g., HOMA-IR), their direct effect on BW remains relatively limited. Furthermore, this phenomenon might be associated with the complex metabolic characteristics inherent to SCH spectrum disorders and the methodological limitations of the study design.

Collectively, our findings hold considerable clinical significance. The results suggested that the probiotic-based gut microbiota modulation may be beneficial for improving psychiatric symptoms and metabolic parameters in SCH. However, the certainty of the evidence is low due to the risk of bias and imprecision. It is recommended that future large-scale, high-quality randomized controlled trials be conducted. These trials should be well-reported and standardize the reporting of strain, dose, metabolic status, and medication background to enable more detailed analyses.

### 4.2 Strengths

This study presents several significant strengths. First, the rigorous implementation of inclusion and exclusion criteria ensured that high-quality and reliable research is conducted. Systematic searches across multiple databases, combined with quality assessments of included studies, helpfully reduced bias and enhanced the credibility of the findings. Second, our analysis incorporated 13 studies involving 783 patients, building upon prior reviews of gut microbiota interventions in SCH by introducing two novel intervention modalities, namely, synbiotics and dietary modifications. Third, the inclusion of 23 outcome indicators across three major categories offers more current and comprehensive evidence-based recommendations compared to prior research. Finally, stratified analysis of different intervention effects provides a clearer distinction of how various approaches impact gut microbiota and clinical symptoms in SCH patients, yielding targeted references for clinical practice.

### 4.3 Limitations

However, several limitations merit consideration. First, the scarcity of included studies presents constraints, particularly evident in the disproportionate representation of interventions: probiotic monotherapy accounted for 90% (9/10) of studies, while research on dietary modifications and synbiotic interventions remained limited (1 study each). This imbalance potentially impedes a comprehensive evaluation of alternative therapeutic approaches. And given the emerging nature of this field and the scarcity of clinical trials, we opted for a broader inclusion approach to allow a more comprehensive synthesis of existing evidence, so we did not impose a strict minimum intervention duration criterion during the study selection process. Second, our review was limited to studies published in English, which may introduce language bias and lead to the omission of potentially relevant data published in other languages. Although we made efforts to search gray literature and trial registries, it is possible that some unpublished studies or those published in non-English journals were missed. These limitations could affect the comprehensiveness and generalizability of our findings. Future meta-analyses would benefit from including non-English literature to provide a more complete picture. Third, geographical bias arises as 10 studies originated from Asian populations, given the recognized regional and dietary cultural specificity of gut microbiota composition. Consequently, the generalizability of conclusions to non-Asian populations necessitates validation through multicenter, large-sample investigations. Forth, insufficient consideration of demographic variables (gender, age) and regional differences in current studies may constrain the interpretive validity. Finally, the complex pathogenesis of SCH and incomplete elucidation of gut microbiota alterations, coupled with the undetermined causal relationship between microbial changes and psychiatric symptoms, demand cautious interpretation of findings. These limitations suggest that, it is necessary to conduct some high-quality RCTs to confirm the current conclusions.

## 5 Conclusion

All in all, after a comprehensive comparison of the above-mentioned outcome indicators of the three different intervention methods, the probiotic-based gut microbiota modulation may offer modest yet promising therapeutic benefits, primarily for improving certain metabolic parameters and positive symptoms. Future large-scale, standardized trials are needed to confirm these preliminary findings and establish definitive clinical efficacy.

## Data Availability

The original contributions presented in the study are included in the article/Supplementary material, further inquiries can be directed to the corresponding author.
