# Correction: Effects of gut microbiota interventions on patients with schizophrenia: a systematic review and meta-analysis

**DOI:** 10.3389/fmicb.2025.1744519

**Published:** 2026-01-12

**Authors:** Nianhua Ye, Xin Song, Jing Yu, Xiaolei Bao, Minghua Ye, Lisheng Jiang

**Affiliations:** 1 College of Traditional Chinese Medicine, Jiangxi University of Chinese Medicine, Nanchang, Jiangxi, China; 2 College of Humanities, Jiangxi University of Chinese Medicine, Nanchang, Jiangxi, China; 3 College of Chinese Classics Studies, Beijing University of Chinese Medicine, Beijing, China

**Keywords:** gut microbiota, probiotics, schizophrenia, effects, meta-analysis

An incorrect **Funding** statement was provided. The funders NATCM, (Grant No. zyyzdxk-2023112) and Jiangxi University of Chinese Medicine (Grant No. 202510412242) were erroneously omitted. The correct **Funding** statement reads:

“The author(s) declared that financial support was received for this work and/or its publication. This work was supported by the General Project on the Compilation of Abstracts of Chinese Medical Collections by the National Administration of Traditional Chinese Medicine (No. KJS-ZHYC-2020-011), the Special Project on the Inheritance of TCM Ancient Documents and Characteristic Techniques in 2021 by the National Administration of Traditional Chinese Medicine (No. GZY-KJS-2021-049) in China, the Project of High-level Construction of Key TCM Disciplines by the National Administration of Traditional Chinese Medicine (No. zyyzdxk-2023112), and the Project of Innovative and Entrepreneurial for College Students by Jiangxi University of Chinese Medicine (No. 202510412242).”

The original version of this article has been updated.

