# Supplementary material for: Effects of gut microbiota interventions on patients with schizophrenia: a systematic review and meta-analysis
Source: Front Microbiol. 2025 Nov 6;16:1681559. doi: 10.3389/fmicb.2025.1681559 (PMC12630112; doi:10.3389/fmicb.2025.1681559)
Supplement: Supplementary file 2 [file Table_2.DOCX]

**Supplement Table S2: The Search Strategy for the Databases Used**

| **Database** | **NO.** | **Search terms** |
| --- | --- | --- |
| PUBMED (101) | #1 | (Schizophrenia[MeSH Terms]) OR (Schizophrenia Spectrum and Other Psychotic Disorders[MeSH Terms]) |
|  | #2 | ((((((((((((((((((((((((((((((((((Schizophrenia) OR (Schizophrenia Spectrum and Other Psychotic Disorders)) OR (Schizophrenias)) OR (Dementia Praecox)) OR (Schizophrenic Disorders)) OR (Disorder, Schizophrenic)) OR (Disorders, Schizophrenic)) OR (Schizophrenic Disorder)) OR (Schizophrenia and Disorders with Psychotic Features)) OR (Paranoid Schizophrenias)) OR (Schizophrenias, Paranoid)) OR (Paranoid Schizophrenia)) OR (Delusional Disorder)) OR (Delusional Disorders)) OR (Disorder, Delusional)) OR (Disorders, Delusional)) OR (Disorganized Schizophrenia)) OR (Disorganized Schizophrenias)) OR (Schizophrenias, Disorganized)) OR (Hebephrenic Schizophrenia)) OR (Hebephrenic Schizophrenias)) OR (Schizophrenias, Hebephrenic)) OR (Schizophrenia, Hebephrenic)) OR (Childhood Schizophrenia)) OR (Childhood-Onset Schizophrenia)) OR (Childhood Onset Schizophrenia)) OR (Schizophrenia, Childhood-Onset)) OR (Catatonic Schizophrenia)) OR (Catatonic Schizophrenias)) OR (Schizophrenias, Catatonic)) OR (Schizophrenia, Treatment Resistant)) OR (Treatment-Resistant Schizophrenia)) OR (Treatment Resistant Schizophrenia)) OR (Refractory Schizophrenia)) OR (Schizophrenia, Refractory) |
|  | #3 | (#1) OR (#2) |
|  | #4 | ((prebiotics[MeSH Terms]) OR (probiotics[MeSH Terms])) OR (diet regulation[MeSH Terms]) |
|  | #5 | ((((((((Fermented food[MeSH Terms]) OR (Fermented Food[MeSH Terms])) OR (Food, Fermented[MeSH Terms])) OR (Foods, Fermented[MeSH Terms])) OR (Fermented Foods and Beverages[MeSH Terms])) OR (Cultured Foods[MeSH Terms])) OR (Cultured Food[MeSH Terms])) OR (Food, Cultured[MeSH Terms])) OR (Foods, Cultured[MeSH Terms]) |
|  | #6 | (((((((((((((((((((((((((((((((((((((((((((((((((((((((((((((((((((((((((fecal microbiota transplantation[MeSH Terms]) OR (Fecal Microbiota Transplantations[MeSH Terms])) OR (Microbiota Transplantation, Fecal[MeSH Terms])) OR (Microbiota Transplantations, Fecal[MeSH Terms])) OR (Transplantation, Fecal Microbiota[MeSH Terms])) OR (Transplantations, Fecal Microbiota[MeSH Terms])) OR (Fecal Microbiota Transplant[MeSH Terms])) OR (Fecal Microbiota Transplants[MeSH Terms])) OR (Microbiota Transplant, Fecal[MeSH Terms])) OR (Microbiota Transplants, Fecal[MeSH Terms])) OR (Transplant, Fecal Microbiota[MeSH Terms])) OR (Transplants, Fecal Microbiota[MeSH Terms])) OR (Fecal Microbiome Transplantation[MeSH Terms])) OR (Fecal Microbiome Transplantations[MeSH Terms])) OR (Microbiome Transplantation, Fecal[MeSH Terms])) OR (Microbiome Transplantations, Fecal[MeSH Terms])) OR (Transplantation, Fecal Microbiome[MeSH Terms])) OR (Transplantations, Fecal Microbiome[MeSH Terms])) OR (Fecal Transplant[MeSH Terms])) OR (Fecal Transplants[MeSH Terms])) OR (Transplant, Fecal[MeSH Terms])) OR (Transplants, Fecal[MeSH Terms])) OR (Donor Feces Infusion[MeSH Terms])) OR (Donor Feces Infusions[MeSH Terms])) OR (Feces Infusion, Donor[MeSH Terms])) OR (Feces Infusions, Donor[MeSH Terms])) OR (Infusion, Donor Feces[MeSH Terms])) OR (Infusions, Donor Feces[MeSH Terms])) OR (Fecal Transplantation[MeSH Terms])) OR (Fecal Transplantations[MeSH Terms])) OR (Transplantation, Fecal[MeSH Terms])) OR (Transplantations, Fecal[MeSH Terms])) OR (Intestinal Microbiota Transfer[MeSH Terms])) OR (Intestinal Microbiota Transfers[MeSH Terms])) OR (Microbiota Transfer, Intestinal[MeSH Terms])) OR (Microbiota Transfers, Intestinal[MeSH Terms])) OR (Transfer, Intestinal Microbiota[MeSH Terms])) OR (Transfers, Intestinal Microbiota[MeSH Terms])) OR (Intestinal Microbiota Transplantation[MeSH Terms])) OR (Intestinal Microbiota Transplantations[MeSH Terms])) OR (Microbiota Transplantation, Intestinal[MeSH Terms])) OR (Microbiota Transplantations, Intestinal[MeSH Terms])) OR (Transplantation, Intestinal Microbiota[MeSH Terms])) OR (Transplantations, Intestinal Microbiota[MeSH Terms])) OR (Intestinal Microbiome Transplantation[MeSH Terms])) OR (Intestinal Microbiome Transplantations[MeSH Terms])) OR (Microbiome Transplantation, Intestinal[MeSH Terms])) OR (Microbiome Transplantations, Intestinal[MeSH Terms])) OR (Transplantation, Intestinal Microbiome[MeSH Terms])) OR (Transplantations, Intestinal Microbiome[MeSH Terms])) OR (Intestinal Microbiota Transplant[MeSH Terms])) OR (Intestinal Microbiota Transplants[MeSH Terms])) OR (Microbiota Transplant, Intestinal[MeSH Terms])) OR (Microbiota Transplants, Intestinal[MeSH Terms])) OR (Transplant, Intestinal Microbiota[MeSH Terms])) OR (Transplants, Intestinal Microbiota[MeSH Terms])) OR (Intestinal Microbiome Transfer[MeSH Terms])) OR (Intestinal Microbiome Transfers[MeSH Terms])) OR (Microbiome Transfer, Intestinal[MeSH Terms])) OR (Microbiome Transfers, Intestinal[MeSH Terms])) OR (Transfer, Intestinal Microbiome[MeSH Terms])) OR (Transfers, Intestinal Microbiome[MeSH Terms])) OR (Fecal Microbiota Transfer[MeSH Terms])) OR (Fecal Microbiota Transfers[MeSH Terms])) OR (Microbiota Transfer, Fecal[MeSH Terms])) OR (Microbiota Transfers, Fecal[MeSH Terms])) OR (Transfer, Fecal Microbiota[MeSH Terms])) OR (Transfers, Fecal Microbiota[MeSH Terms])) OR (Intestinal Microbiome Transplant[MeSH Terms])) OR (Intestinal Microbiome Transplants[MeSH Terms])) OR (Microbiome Transplant, Intestinal[MeSH Terms])) OR (Microbiome Transplants, Intestinal[MeSH Terms])) OR (Transplant, Intestinal Microbiome[MeSH Terms])) OR (Transplants, Intestinal Microbiome[MeSH Terms]) |
|  | #7 | ((#4) OR (#5)) OR (#6) |
|  | #8 | (#3) AND (#7) (**Filters: Clinical Trial, Randomized Controlled Trial)** |
| Embase (806) | #1 | 'schizophrenia'/exp OR schizophrenia OR (('schizophrenia spectrum' OR (('schizophrenia'/exp OR schizophrenia) AND ('spectrum'/exp OR spectrum))) AND ('other psychotic disorders' OR (other AND ('psychotic'/exp OR psychotic) AND ('disorders'/exp OR disorders)))) OR schizophrenias OR 'dementia praecox'/exp OR 'dementia praecox' OR (('dementia'/exp OR dementia) AND praecox) OR 'schizophrenic disorders' OR (('schizophrenic'/exp OR schizophrenic) AND ('disorders'/exp OR disorders)) OR 'disorder, schizophrenic' OR (('disorder,'/exp OR disorder,) AND ('schizophrenic'/exp OR schizophrenic)) OR 'disorders, schizophrenic' OR (('disorders,'/exp OR disorders,) AND ('schizophrenic'/exp OR schizophrenic)) OR 'schizophrenic disorder' OR (('schizophrenic'/exp OR schizophrenic) AND ('disorder'/exp OR disorder)) OR (('schizophrenia'/exp OR schizophrenia) AND ('disorders with psychotic features' OR (('disorders'/exp OR disorders) AND with AND ('psychotic'/exp OR psychotic) AND features))) OR 'paranoid schizophrenias' OR (('paranoid'/exp OR paranoid) AND schizophrenias) OR 'schizophrenias, paranoid' OR (schizophrenias, AND ('paranoid'/exp OR paranoid)) OR paranoid schizophrenia OR 'delusional disorder'/exp OR 'delusional disorder' OR (delusional AND ('disorder'/exp OR disorder)) OR 'delusional disorders' OR (delusional AND ('disorders'/exp OR disorders)) OR 'disorder, delusional' OR (('disorder,'/exp OR disorder,) AND delusional) OR 'disorders, delusional' OR (('disorders,'/exp OR disorders,) AND delusional) OR disorganized schizophrenia OR 'disorganized schizophrenias' OR (disorganized AND schizophrenias) OR 'schizophrenias, disorganized' OR (schizophrenias, AND disorganized) OR hebephrenic schizophrenia OR 'hebephrenic schizophrenias' OR (hebephrenic AND schizophrenias) OR 'schizophrenias, hebephrenic' OR (schizophrenias, AND hebephrenic) OR 'schizophrenia, hebephrenic'/exp OR 'schizophrenia, hebephrenic' OR (('schizophrenia,'/exp OR schizophrenia,) AND hebephrenic) OR 'childhood schizophrenia'/exp OR 'childhood schizophrenia' OR (('childhood'/exp OR childhood) AND ('schizophrenia'/exp OR schizophrenia)) OR 'childhood-onset schizophrenia' OR ('childhood onset' AND ('schizophrenia'/exp OR schizophrenia)) OR 'childhood onset schizophrenia'/exp OR 'childhood onset schizophrenia' OR (('childhood'/exp OR childhood) AND onset AND ('schizophrenia'/exp OR schizophrenia)) OR 'schizophrenia, childhood-onset' OR (('schizophrenia,'/exp OR schizophrenia,) AND 'childhood onset') OR 'catatonic schizophrenia'/exp OR 'catatonic schizophrenia' OR (catatonic AND ('schizophrenia'/exp OR schizophrenia)) OR 'catatonic schizophrenias' OR (catatonic AND schizophrenias) OR 'schizophrenias, catatonic' OR (schizophrenias, AND catatonic) OR 'schizophrenia, treatment resistant'/exp OR 'schizophrenia, treatment resistant' OR (('schizophrenia,'/exp OR schizophrenia,) AND ('treatment'/exp OR treatment) AND resistant) OR 'treatment-resistant schizophrenia'/exp OR 'treatment-resistant schizophrenia' OR ('treatment resistant' AND ('schizophrenia'/exp OR schizophrenia)) OR 'treatment resistant schizophrenia'/exp OR 'treatment resistant schizophrenia' OR (('treatment'/exp OR treatment) AND resistant AND ('schizophrenia'/exp OR schizophrenia)) OR 'refractory schizophrenia'/exp OR 'refractory schizophrenia' OR (refractory AND ('schizophrenia'/exp OR schizophrenia)) OR 'schizophrenia, refractory' OR (('schizophrenia,'/exp OR schizophrenia,) AND refractory) |
|  | #2 | 'probiotics'/exp OR probiotics OR 'prebiotics'/exp OR prebiotics OR 'fecal microbiota transplantation'/exp OR 'fecal microbiota transplantation' OR (fecal AND ('microbiota'/exp OR microbiota) AND ('transplantation'/exp OR transplantation)) OR biostime OR 'postbiotics'/exp OR postbiotics OR 'diet regulation' OR (('diet'/exp OR diet) AND ('regulation'/exp OR regulation)) OR 'microbiota transplantation, fecal' OR (('microbiota'/exp OR microbiota) AND ('transplantation,'/exp OR transplantation,) AND fecal) OR 'microbiota transplantations, fecal' OR (('microbiota'/exp OR microbiota) AND transplantations, AND fecal) OR 'transplantation, fecal microbiota' OR (('transplantation,'/exp OR transplantation,) AND fecal AND ('microbiota'/exp OR microbiota)) OR 'transplantations, fecal microbiota' OR (transplantations, AND fecal AND ('microbiota'/exp OR microbiota)) OR 'fecal microbiota transplant'/exp OR 'fecal microbiota transplant' OR (fecal AND ('microbiota'/exp OR microbiota) AND ('transplant'/exp OR transplant)) OR 'microbiota transplant, fecal' OR (('microbiota'/exp OR microbiota) AND transplant, AND fecal) OR 'microbiota transplants, fecal' OR (('microbiota'/exp OR microbiota) AND ('transplants,'/exp OR transplants,) AND fecal) OR 'transplant, fecal microbiota' OR (transplant, AND fecal AND ('microbiota'/exp OR microbiota)) OR 'transplants, fecal microbiota' OR (('transplants,'/exp OR transplants,) AND fecal AND ('microbiota'/exp OR microbiota)) OR 'fecal microbiome transplantation'/exp OR 'fecal microbiome transplantation' OR (fecal AND ('microbiome'/exp OR microbiome) AND ('transplantation'/exp OR transplantation)) OR 'fecal microbiome transplantations' OR (fecal AND ('microbiome'/exp OR microbiome) AND transplantations) OR 'microbiome transplantation, fecal' OR (('microbiome'/exp OR microbiome) AND ('transplantation,'/exp OR transplantation,) AND fecal) OR 'microbiome transplantations, fecal' OR (('microbiome'/exp OR microbiome) AND transplantations, AND fecal) OR 'transplantation, fecal microbiome' OR (('transplantation,'/exp OR transplantation,) AND fecal AND ('microbiome'/exp OR microbiome)) OR 'transplantations, fecal microbiome' OR (transplantations, AND fecal AND ('microbiome'/exp OR microbiome)) OR 'fecal transplant'/exp OR 'fecal transplant' OR (fecal AND ('transplant'/exp OR transplant)) OR 'fecal transplants' OR (fecal AND ('transplants'/exp OR transplants)) OR 'transplants, fecal' OR (('transplants,'/exp OR transplants,) AND fecal) OR 'donor feces infusion' OR (('donor'/exp OR donor) AND ('feces'/exp OR feces) AND ('infusion'/exp OR infusion)) OR 'transplant, fecal' OR (transplant, AND fecal) OR 'donor feces infusions' OR (('donor'/exp OR donor) AND ('feces'/exp OR feces) AND infusions) OR 'feces infusion, donor' OR (('feces'/exp OR feces) AND ('infusion,'/exp OR infusion,) AND ('donor'/exp OR donor)) OR 'feces infusions, donor' OR (('feces'/exp OR feces) AND infusions, AND ('donor'/exp OR donor)) OR 'infusion, donor feces' OR (('infusion,'/exp OR infusion,) AND ('donor'/exp OR donor) AND ('feces'/exp OR feces)) OR 'infusions, donor feces' OR (infusions, AND ('donor'/exp OR donor) AND ('feces'/exp OR feces)) OR 'fecal transplantation'/exp OR 'fecal transplantation' OR (fecal AND ('transplantation'/exp OR transplantation)) OR 'fecal transplantations' OR (fecal AND transplantations) OR 'transplantation, fecal' OR (('transplantation,'/exp OR transplantation,) AND fecal) OR 'fermented food'/exp OR 'fermented food' OR (fermented AND ('food'/exp OR food)) OR 'foods, fermented' OR (foods, AND fermented) OR (('fermented foods'/exp OR 'fermented foods' OR (fermented AND foods)) AND ('beverages'/exp OR beverages)) OR 'cultured foods' OR (cultured AND foods) OR 'cultured food' OR (cultured AND ('food'/exp OR food)) OR 'food, cultured' OR (('food,'/exp OR food,) AND cultured) OR 'foods, cultured' OR (foods, AND cultured) OR 'transplantations, fecal' OR (transplantations, AND fecal) OR 'intestinal microbiota transfer' OR (intestinal AND ('microbiota'/exp OR microbiota) AND ('transfer'/exp OR transfer)) OR 'intestinal microbiota transfers' OR (intestinal AND ('microbiota'/exp OR microbiota) AND transfers) OR 'microbiota transfer, intestinal' OR (('microbiota'/exp OR microbiota) AND transfer, AND intestinal) OR 'microbiota transfers, intestinal' OR (('microbiota'/exp OR microbiota) AND transfers, AND intestinal) OR 'transfer, intestinal microbiota' OR (transfer, AND intestinal AND ('microbiota'/exp OR microbiota)) OR 'transfers, intestinal microbiota' OR (transfers, AND intestinal AND ('microbiota'/exp OR microbiota)) OR 'intestinal microbiota transplantation'/exp OR 'intestinal microbiota transplantation' OR (intestinal AND ('microbiota'/exp OR microbiota) AND ('transplantation'/exp OR transplantation)) OR 'intestinal microbiota transplantations' OR (intestinal AND ('microbiota'/exp OR microbiota) AND transplantations) OR 'microbiota transplantation, intestinal' OR (('microbiota'/exp OR microbiota) AND ('transplantation,'/exp OR transplantation,) AND intestinal) OR 'microbiota transplantations, intestinal' OR (('microbiota'/exp OR microbiota) AND transplantations, AND intestinal) OR 'transplantation, intestinal microbiota' OR (('transplantation,'/exp OR transplantation,) AND intestinal AND ('microbiota'/exp OR microbiota)) OR 'transplantations, intestinal microbiota' OR (transplantations, AND intestinal AND ('microbiota'/exp OR microbiota)) OR 'intestinal microbiome transplantation' OR (intestinal AND ('microbiome'/exp OR microbiome) AND ('transplantation'/exp OR transplantation)) OR 'intestinal microbiome transplantations' OR (intestinal AND ('microbiome'/exp OR microbiome) AND transplantations) OR 'microbiome transplantation, intestinal' OR (('microbiome'/exp OR microbiome) AND ('transplantation,'/exp OR transplantation,) AND intestinal) OR 'microbiome transplantations, intestinal' OR (('microbiome'/exp OR microbiome) AND transplantations, AND intestinal) OR 'transplantation, intestinal microbiome' OR (('transplantation,'/exp OR transplantation,) AND intestinal AND ('microbiome'/exp OR microbiome)) OR 'transplantations, intestinal microbiome' OR (transplantations, AND intestinal AND ('microbiome'/exp OR microbiome)) OR 'intestinal microbiota transplant'/exp OR 'intestinal microbiota transplant' OR (intestinal AND ('microbiota'/exp OR microbiota) AND ('transplant'/exp OR transplant)) OR 'intestinal microbiota transplants' OR (intestinal AND ('microbiota'/exp OR microbiota) AND ('transplants'/exp OR transplants)) OR 'microbiota transplant, intestinal' OR (('microbiota'/exp OR microbiota) AND transplant, AND intestinal) OR 'microbiota transplants, intestinal' OR (('microbiota'/exp OR microbiota) AND ('transplants,'/exp OR transplants,) AND intestinal) OR 'transplant, intestinal microbiota' OR (transplant, AND intestinal AND ('microbiota'/exp OR microbiota)) OR 'transplants, intestinal microbiota' OR (('transplants,'/exp OR transplants,) AND intestinal AND ('microbiota'/exp OR microbiota)) OR 'intestinal microbiome transfer' OR (intestinal AND ('microbiome'/exp OR microbiome) AND ('transfer'/exp OR transfer)) OR 'intestinal microbiome transfers' OR (intestinal AND ('microbiome'/exp OR microbiome) AND transfers) OR 'microbiome transfer, intestinal' OR (('microbiome'/exp OR microbiome) AND transfer, AND intestinal) OR 'microbiome transfers, intestinal' OR (('microbiome'/exp OR microbiome) AND transfers, AND intestinal) OR 'transfer, intestinal microbiome' OR (transfer, AND intestinal AND ('microbiome'/exp OR microbiome)) OR 'transfers, intestinal microbiome' OR (transfers, AND intestinal AND ('microbiome'/exp OR microbiome)) OR 'fecal microbiota transfer' OR (fecal AND ('microbiota'/exp OR microbiota) AND ('transfer'/exp OR transfer)) OR 'fecal microbiota transfers' OR (fecal AND ('microbiota'/exp OR microbiota) AND transfers) OR 'microbiota transfer, fecal' OR (('microbiota'/exp OR microbiota) AND transfer, AND fecal) OR 'microbiota transfers, fecal' OR (('microbiota'/exp OR microbiota) AND transfers, AND fecal) OR 'transfer, fecal microbiota' OR (transfer, AND fecal AND ('microbiota'/exp OR microbiota)) OR 'transfers, fecal microbiota' OR (transfers, AND fecal AND ('microbiota'/exp OR microbiota)) OR 'intestinal microbiome transplant' OR (intestinal AND ('microbiome'/exp OR microbiome) AND ('transplant'/exp OR transplant)) OR 'intestinal microbiome transplants' OR (intestinal AND ('microbiome'/exp OR microbiome) AND ('transplants'/exp OR transplants)) OR 'microbiome transplant, intestinal' OR (('microbiome'/exp OR microbiome) AND transplant, AND intestinal) OR 'microbiome transplants, intestinal' OR (('microbiome'/exp OR microbiome) AND ('transplants,'/exp OR transplants,) AND intestinal) OR 'transplant, intestinal microbiome' OR (transplant, AND intestinal AND ('microbiome'/exp OR microbiome)) OR 'transplants, intestinal microbiome' OR (('transplants,'/exp OR transplants,) AND intestinal AND ('microbiome'/exp OR microbiome)) |
|  | #3 | #1 AND #2 **(No filters)** |
| WOS (411) | #1 | ((((((((((((((((((((((((((((((((((ALL=(Schizophrenia )) OR ALL=(Schizophrenia Spectrum and Other Psychotic Disorders)) OR ALL=(Schizophrenias)) OR ALL=(Schizophrenias)) OR ALL=(Schizophrenic Disorders)) OR ALL=(Disorder, Schizophrenic)) OR ALL=(Disorders, Schizophrenic)) OR ALL=(Schizophrenic Disorder)) OR ALL=(Schizophrenia and Disorders with Psychotic Features)) OR ALL=(Paranoid Schizophrenias)) OR ALL=(Schizophrenias, Paranoid)) OR ALL=(Paranoid Schizophrenia)) OR ALL=(Delusional Disorder)) OR ALL=(Delusional Disorders)) OR ALL=(Disorder, Delusional)) OR ALL=(Disorders, Delusional)) OR ALL=(Disorganized Schizophrenia)) OR ALL=(Disorganized Schizophrenias)) OR ALL=(Schizophrenias, Disorganized)) OR ALL=(Hebephrenic Schizophrenia)) OR ALL=(Hebephrenic Schizophrenias)) OR ALL=(Schizophrenias, Hebephrenic)) OR ALL=(Schizophrenia, Hebephrenic)) OR ALL=(Childhood Schizophrenia)) OR ALL=(Childhood-Onset Schizophrenia)) OR ALL=(Childhood Onset Schizophrenia)) OR ALL=(Schizophrenia, Childhood-Onset)) OR ALL=(Catatonic Schizophrenia)) OR ALL=(Catatonic Schizophrenias)) OR ALL=(Schizophrenias, Catatonic)) OR ALL=(Schizophrenia, Treatment Resistant)) OR ALL=(Treatment-Resistant Schizophrenia)) OR ALL=(Treatment Resistant Schizophrenia)) OR ALL=(Refractory Schizophrenia)) OR ALL=(Schizophrenia, Refractory) |
|  | #2 | (((((((((((((((((((((((((((((((((((((((((((((((((((((((((((((((((((((((((((((((((((((((TS=(probiotics)) OR TS=(prebiotics)) OR TS=(postbiotics)) OR TS=(diet regulation)) OR TS=(synbiotics)) OR TS=(fecal microbiota transplantation)) OR TS=(Fecal Microbiota Transplantations)) OR TS=(Microbiota Transplantation, Fecal)) OR TS=(Microbiota Transplantations, Fecal)) OR TS=(Transplantation, Fecal Microbiota)) OR TS=(Transplantations, Fecal Microbiota)) OR TS=(Fecal Microbiota Transplant)) OR TS=(Fecal Microbiota Transplants)) OR TS=(Microbiota Transplant, Fecal)) OR TS=(Microbiota Transplants, Fecal)) OR TS=(Transplant, Fecal Microbiota)) OR TS=(Transplants, Fecal Microbiota)) OR TS=(Fecal Microbiome Transplantation)) OR TS=(Fecal Microbiome Transplantations)) OR TS=(Microbiome Transplantation, Fecal)) OR TS=(Microbiome Transplantations, Fecal)) OR TS=(Transplantation, Fecal Microbiome)) OR TS=(Transplantations, Fecal Microbiome)) OR TS=(Fecal Transplant)) OR TS=(Fecal Transplants)) OR TS=(Transplant, Fecal)) OR TS=(Transplants, Fecal)) OR TS=(Donor Feces Infusion)) OR TS=(Donor Feces Infusions)) OR TS=(Feces Infusion, Donor)) OR TS=(Feces Infusions, Donor)) OR TS=(Infusion, Donor Feces)) OR TS=(Infusions, Donor Feces)) OR TS=(Fecal Transplantation)) OR TS=(Fecal Transplantations)) OR TS=(Transplantation, Fecal)) OR TS=(Transplantations, Fecal)) OR TS=(Intestinal Microbiota Transfer)) OR TS=(Intestinal Microbiota Transfers)) OR TS=(Microbiota Transfer, Intestinal)) OR TS=(Microbiota Transfers, Intestinal)) OR TS=(Transfer, Intestinal Microbiota)) OR TS=(Transfers, Intestinal Microbiota)) OR TS=(Intestinal Microbiota Transplantation)) OR TS=(Intestinal Microbiota Transplantations)) OR TS=(Microbiota Transplantation, Intestinal)) OR TS=(Microbiota Transplantations, Intestinal)) OR TS=(Transplantation, Intestinal Microbiota)) OR TS=(Transplantations, Intestinal Microbiota)) OR TS=(Intestinal Microbiome Transplantation)) OR TS=(Intestinal Microbiome Transplantations)) OR TS=(Microbiome Transplantation, Intestinal)) OR TS=(Microbiome Transplantations, Intestinal)) OR TS=(Transplantation, Intestinal Microbiome)) OR TS=(Transplantations, Intestinal Microbiome)) OR TS=(Intestinal Microbiota Transplant)) OR TS=(Intestinal Microbiota Transplants)) OR TS=(Microbiota Transplant, Intestinal)) OR TS=(Microbiota Transplants, Intestinal)) OR TS=(Transplant, Intestinal Microbiota)) OR TS=(Transplants, Intestinal Microbiota)) OR TS=(Intestinal Microbiome Transfer)) OR TS=(Intestinal Microbiome Transfers)) OR TS=(Microbiome Transfer, Intestinal)) OR TS=(Microbiome Transfers, Intestinal)) OR TS=(Transfer, Intestinal Microbiome)) OR TS=(Transfers, Intestinal Microbiome)) OR TS=(Fecal Microbiota Transfer)) OR TS=(Fecal Microbiota Transfers)) OR TS=(Microbiota Transfer, Fecal)) OR TS=(Microbiota Transfers, Fecal)) OR TS=(Transfer, Fecal Microbiota)) OR TS=(Transfers, Fecal Microbiota)) OR TS=(Intestinal Microbiome Transplant)) OR TS=(Intestinal Microbiome Transplants)) OR TS=(Microbiome Transplant, Intestinal)) OR TS=(Microbiome Transplants, Intestinal)) OR TS=(Transplant, Intestinal Microbiome)) OR TS=(Transplants, Intestinal Microbiome)) OR TS=(Fermented food)) OR TS=(Fermented Food)) OR TS=(Food, Fermented)) OR TS=(Foods, Fermented)) OR TS=(Fermented Foods and Beverages)) OR TS=(Cultured Foods)) OR TS=(Cultured Food)) OR TS=(Food, Cultured)) OR TS=(Foods, Cultured) and Preprint Citation Index (Exclude – Database) |
|  | #3 | #1 AND #2 **(No filters)** |
| Cochrane (53) | #1 | (Schizophrenia):ti,ab,kw OR (Schizophrenia Spectrum and Other Psychotic Disorders):ti,ab,kw OR (Schizophrenias):ti,ab,kw OR (Dementia Praecox):ti,ab,kw OR (Schizophrenic Disorders):ti,ab,kw (Word variations have been searched) |
|  | #2 | (Disorder, Schizophrenic):ti,ab,kw OR (Disorders, Schizophrenic):ti,ab,kw OR (Schizophrenic Disorder):ti,ab,kw OR (Schizophrenia and Disorders with Psychotic Features):ti,ab,kw OR (Paranoid Schizophrenias):ti,ab,kw (Word variations have been searched) |
|  | #3 | (Schizophrenias, Paranoid):ti,ab,kw OR (Paranoid Schizophrenia):ti,ab,kw OR (Delusional Disorder):ti,ab,kw OR (Delusional Disorders):ti,ab,kw OR (Disorder, Delusional):ti,ab,kw (Word variations have been searched) |
|  | #4 | (Disorders, Delusional):ti,ab,kw OR (Disorganized Schizophrenia):ti,ab,kw OR (Disorganized Schizophrenias):ti,ab,kw OR (Schizophrenias, Disorganized):ti,ab,kw OR (Hebephrenic Schizophrenia):ti,ab,kw (Word variations have been searched) |
|  | #5 | (Hebephrenic Schizophrenias):ti,ab,kw OR (Schizophrenias, Hebephrenic):ti,ab,kw OR (Schizophrenia, Hebephrenic):ti,ab,kw OR (Childhood Schizophrenia):ti,ab,kw OR (Childhood-Onset Schizophrenia):ti,ab,kw (Word variations have been searched) |
|  | #6 | (Childhood Onset Schizophrenia):ti,ab,kw OR (Schizophrenia, Childhood-Onset):ti,ab,kw OR (Catatonic Schizophrenia):ti,ab,kw OR (Catatonic Schizophrenias):ti,ab,kw OR (Schizophrenias, Catatonic):ti,ab,kw (Word variations have been searched) |
|  | #7 | (Schizophrenia, Treatment Resistant):ti,ab,kw OR (Treatment-Resistant Schizophrenia):ti,ab,kw OR (Treatment Resistant Schizophrenia):ti,ab,kw OR (Refractory Schizophrenia):ti,ab,kw OR (Schizophrenia, Refractory):ti,ab,kw (Word variations have been searched) |
|  | #8 | (#1) OR (#2) OR (#3) OR (#4) OR (#5) OR (#6) OR (#7) |
|  | #9 | (probiotics):ti,ab,kw OR (probiotic):ti,ab,kw OR (prebiotics):ti,ab,kw OR (synbiotics):ti,ab,kw OR (synbiotic):ti,ab,kw (Word variations have been searched) |
|  | #10 | (Fermented Foods and Beverages):ti,ab,kw OR (Cultured Foods):ti,ab,kw OR (Cultured Food):ti,ab,kw OR (Food, Cultured):ti,ab,kw OR (Foods, Cultured):ti,ab,kw (Word variations have been searched) |
|  | #11 | (Transplantations, Fecal Microbiota):ti,ab,kw OR (Fecal Microbiota Transplant):ti,ab,kw OR (Fecal Microbiota Transplants):ti,ab,kw OR (Microbiota Transplant, Fecal):ti,ab,kw OR (Microbiota Transplants, Fecal):ti,ab,kw (Word variations have been searched) |
|  | #12 | (Transplant, Fecal Microbiota):ti,ab,kw OR (Transplants, Fecal Microbiota):ti,ab,kw OR (Fecal Microbiome Transplantation):ti,ab,kw OR (Fecal Microbiome Transplantations):ti,ab,kw OR (Microbiome Transplantation, Fecal):ti,ab,kw (Word variations have been searched) |
|  | #13 | (Microbiome Transplantations, Fecal):ti,ab,kw OR (Transplantation, Fecal Microbiome):ti,ab,kw OR (Transplantations, Fecal Microbiome):ti,ab,kw OR (Fecal Transplant):ti,ab,kw OR (Fecal Transplants):ti,ab,kw (Word variations have been searched) |
|  | #14 | (Transplant, Fecal):ti,ab,kw OR (Transplants, Fecal):ti,ab,kw OR (Donor Feces Infusion):ti,ab,kw OR (Donor Feces Infusions):ti,ab,kw OR (Feces Infusion, Donor):ti,ab,kw (Word variations have been searched) |
|  | #15 | (Feces Infusions, Donor):ti,ab,kw OR (Infusion, Donor Feces):ti,ab,kw OR (Infusions, Donor Feces):ti,ab,kw OR (Fecal Transplantation):ti,ab,kw OR (Fecal Transplantations):ti,ab,kw (Word variations have been searched) |
|  | #16 | (Transplantation, Fecal):ti,ab,kw OR (Transplantations, Fecal):ti,ab,kw OR (Intestinal Microbiota Transfer):ti,ab,kw OR (Intestinal Microbiota Transfers):ti,ab,kw OR (Microbiota Transfer, Intestinal):ti,ab,kw (Word variations have been searched) |
|  | #17 | (Microbiota Transfers, Intestinal):ti,ab,kw OR (Transfer, Intestinal Microbiota):ti,ab,kw OR (Transfers, Intestinal Microbiota):ti,ab,kw OR (Intestinal Microbiota Transplantation):ti,ab,kw OR (Intestinal Microbiota Transplantations):ti,ab,kw (Word variations have been searched) |
|  | #18 | (Microbiota Transplantation, Intestinal):ti,ab,kw OR (Microbiota Transplantations, Intestinal):ti,ab,kw OR (Transplantation, Intestinal Microbiota):ti,ab,kw OR (Transplantations, Intestinal Microbiota):ti,ab,kw OR (Intestinal Microbiome Transplantation):ti,ab,kw (Word variations have been searched) |
|  | #19 | (Intestinal Microbiome Transplantations):ti,ab,kw OR (Microbiome Transplantation, Intestinal):ti,ab,kw OR (Microbiome Transplantations, Intestinal):ti,ab,kw OR (Transplantation, Intestinal Microbiome):ti,ab,kw OR (Transplantations, Intestinal Microbiome):ti,ab,kw (Word variations have been searched) |
|  | #20 | (Intestinal Microbiota Transplant):ti,ab,kw OR (Intestinal Microbiota Transplants):ti,ab,kw OR (Microbiota Transplant, Intestinal):ti,ab,kw OR (Microbiota Transplants, Intestinal):ti,ab,kw OR (Transplant, Intestinal Microbiota):ti,ab,kw (Word variations have been searched) |
|  | #21 | (Transplants, Intestinal Microbiota):ti,ab,kw OR (Intestinal Microbiome Transfer):ti,ab,kw OR (Intestinal Microbiome Transfers):ti,ab,kw OR (Microbiome Transfer, Intestinal):ti,ab,kw OR (Microbiome Transfers, Intestinal):ti,ab,kw (Word variations have been searched) |
|  | #22 | (Transfer, Intestinal Microbiome):ti,ab,kw OR (Transfers, Intestinal Microbiome):ti,ab,kw OR (Fecal Microbiota Transfers):ti,ab,kw OR (Fecal Microbiota Transfer):ti,ab,kw OR (Microbiota Transfer, Fecal):ti,ab,kw (Word variations have been searched) |
|  | #23 | (Microbiome Transplant, Intestinal):ti,ab,kw OR (Microbiome Transplants, Intestinal):ti,ab,kw OR (Transplant, Intestinal Microbiome):ti,ab,kw OR (Transplants, Intestinal Microbiome):ti,ab,kw (Word variations have been searched) |
|  | #24 | (#9) OR (#10) OR (#11) OR (#12) OR (#13) OR (#14) OR (#15) OR (#16) OR (#17) OR (#18) OR (#19) OR (#20) OR (#21) OR (#22) OR (#23) |
|  | #25 | (#8) AND (#24) **(No filters)** |
