# Supplementary material for: Effects of gut microbiota interventions on patients with schizophrenia: a systematic review and meta-analysis
Source: Front Microbiol. 2025 Nov 6;16:1681559. doi: 10.3389/fmicb.2025.1681559 (PMC12630112; doi:10.3389/fmicb.2025.1681559)
Supplement: Supplementary file 3 [file Table_3.DOCX]

**Supplement Table S3: Data Extraction of Outcomes in the Meta-Analysis**

| **NO.** | **Outcomes** | **Study** | **Intervention Group** | | | **Control Group** | | | **Time point** | **Strain & daily dose** |
| --- | --- | --- | --- | --- | --- | --- | --- | --- | --- | --- |
|  |  |  | **n** | **mean** | **sd** | **n** | **mean** | **sd** |  |  |
| 1 | PANSS | Aida Mohammadi 2024 | 35 | -12.9 | 14.4 | 35 | -1.3 | 10.5 | 12 weeks | Lactobacillus acidophilus, L. rhamnosus, L. reuteri, L. paracasei, Bifidobacterium longum, Bacillus coagulans,2×10⁹ CFU |
|  |  | Amir Ghaderi 2019 | 30 | -7.4 | 8.7 | 30 | -1.9 | 7.5 | 12 weeks | L. acidophilus, B. bifidum, L. reuteri, L. fermentum 8×10⁹ CFU/day |
|  |  | Edy Husnul Mujahid 2022 | 21 | -25.8 | 5.4 | 21 | -14.8 | 8.6 | 6 weeks | NA |
|  |  | Faith B Dickerson 2014 | 33 | -4.5 | 10.2 | 32 | -2.5 | 10.3 | 14 weeks | Lactobacillus rhamnosus GG + Bifidobacterium animalis subsp. lactis Bb12, each 10⁹ CFU/day |
|  |  | Hamidreza Jamilian 2021 | 25 | -3.3 | 10.6 | 26 | -1.4 | 10.0 | 12 weeks | L. acidophilus, B. lactis, B. bifidum, B. longum, 8×10⁹ CFU/day |
|  |  | Robabeh Soleimani 2023 | 28 | -26.3 | 11.0 | 27 | -21.1 | 12.7 | 12 weeks | L. acidophilus, L. casei, L. bulgaricus, L. rhamnosus, B. longum, B. breve, S. salivarius, 9×10⁹ CFU/g |
|  |  | Ye Yang 2021 | 33 | -24.3 | 7.8 | 34 | -23.4 | 7.9 | 12 weeks | Bifidobacterium, Lactobacillus, and Enterococcus capsules, 1×10⁷ CFU |
| 2 | BPRS | Amir Ghaderi 2019 | 30 | 0 | 10.15 | 30 | 0.4 | 8.87 | 12 weeks | L. acidophilus, B. bifidum, L. reuteri, L. fermentum 8×10⁹ CFU/day |
|  |  | Hamidreza Jamilian 2021 | 25 | -1.3 | 8.8 | 26 | -0.4 | 9.2 | 12 weeks | L. acidophilus, B. lactis, B. bifidum, B. longum, 8×10⁹ CFU/day |
|  |  | Robabeh Soleimani 2023 | 28 | -16 | 10.4 | 27 | -11.8 | 9.2 | 12 weeks | L. acidophilus, L. casei, L. bulgaricus, L. rhamnosus, B. longum, B. breve, S. salivarius, 9×10⁹ CFU/g |
| 3 | FBS | (A)Jing Huang 2022 | 39 | 0.27 | 0.49 | 37 | 0.16 | 0.31 | 12 weeks | Bifidobacterium, Lactobacillus, Enterococcus (Bifico), 840 mg |
|  |  | Aida Mohammadi 2024 | 35 | -8.49 | 16.42 | 35 | 1.89 | 11.32 | 12 weeks | Lactobacillus acidophilus, L. rhamnosus, L. reuteri, L. paracasei, Bifidobacterium longum, Bacillus coagulans,2×10⁹ CFU |
|  |  | Alfonso Sevillano-Jiménez 2022 | 23 | 3.8 | 12.25 | 21 | 8.1 | 17.94 | 6 months | NA |
|  |  | Amir Ghaderi 2019 | 30 | -7 | 10.87 | 30 | -0.2 | 9.07 | 12 weeks | L. acidophilus, B. bifidum, L. reuteri, L. fermentum 8×10⁹ CFU/day |
|  |  | Hamidreza Jamilian 2021 | 25 | -8.3 | 8.1 | 26 | -0.5 | 8.2 | 12 weeks | L. acidophilus, B. lactis, B. bifidum, B. longum, 8×10⁹ CFU/day |
|  |  | Poorya Basafa-Roodi 2024 | 27 | -5 | 25.6 | 28 | 1 | 18.9 | 8 weeks | L. rhamnosus, L. casei, L. acidophilus, L. bulgaricus, L. plantarum, L. gasseri, L. helveticus, B. lactis, B. breve, B. longum, B. bifidum, S. thermophilus + FOS, Each capsule contains 10⁹ CFU. |
|  |  | Robabeh Soleimani 2023 | 28 | 2.7 | 8.2 | 27 | 5.9 | 9.3 | 12 weeks | L. acidophilus, L. casei, L. bulgaricus, L. rhamnosus, B. longum, B. breve, S. salivarius, 9×10⁹ CFU/g |
| 4 | INS | (A)Jing Huang 2022 | 29 | 1.03 | 12.4 | 28 | 4.38 | 12.8 | 12 weeks | Bifidobacterium, Lactobacillus, Enterococcus (Bifico), 840 mg |
|  |  | Amir Ghaderi 2019 | 30 | -2.7 | 3.7 | 30 | 0.4 | 2.43 | 12 weeks | L. acidophilus, B. bifidum, L. reuteri, L. fermentum 8×10⁹ CFU/day |
|  |  | Hamidreza Jamilian 2021 | 25 | -1.4 | 2.4 | 26 | -0.2 | 2.9 | 12 weeks | L. acidophilus, B. lactis, B. bifidum, B. longum, 8×10⁹ CFU/day |
|  |  | Poorya Basafa-Roodi 2024 | 27 | -2.3 | 15.1 | 28 | -0.2 | 16.8 | 8 weeks | L. rhamnosus, L. casei, L. acidophilus, L. bulgaricus, L. plantarum, L. gasseri, L. helveticus, B. lactis, B. breve, B. longum, B. bifidum, S. thermophilus + FOS, Each capsule contains 10⁹ CFU. |
|  |  | Robabeh Soleimani 2023 | 28 | 0.5 | 1.4 | 28 | -0.2 | 2.9 | 12 weeks | L. acidophilus, L. casei, L. bulgaricus, L. rhamnosus, B. longum, B. breve, S. salivarius, 9×10⁹ CFU/g |
| 5 | TG | (A)Jing Huang 2022 | 39 | 0.44 | 0.28 | 37 | 0.54 | 0.46 | 12 weeks | Bifidobacterium, Lactobacillus, Enterococcus (Bifico), 840 mg |
|  |  | Aida Mohammadi 2024 | 35 | 0.27 | 5.14 | 35 | 0.54 | 6.64 | 12 weeks | Lactobacillus acidophilus, L. rhamnosus, L. reuteri, L. paracasei, Bifidobacterium longum, Bacillus coagulans,2×10⁹ CFU |
|  |  | Alfonso Sevillano-Jiménez 2022 | 23 | -6.9 | 71.98 | 21 | 16.4 | 71.97 | 6 months | NA |
|  |  | Amir Ghaderi 2019 | 30 | -7.8 | 25.2 | 30 | 10.1 | 30.8 | 12 weeks | L. acidophilus, B. bifidum, L. reuteri, L. fermentum 8×10⁹ CFU/day |
|  |  | Hamidreza Jamilian 2021 | 25 | 6.2 | 51.45 | 26 | 3.7 | 43.47 | 12 weeks | L. acidophilus, B. lactis, B. bifidum, B. longum, 8×10⁹ CFU/day |
|  |  | Poorya Basafa-Roodi 2024 | 27 | -46 | 128.27 | 28 | 3 | 115.40 | 8 weeks | L. rhamnosus, L. casei, L. acidophilus, L. bulgaricus, L. plantarum, L. gasseri, L. helveticus, B. lactis, B. breve, B. longum, B. bifidum, S. thermophilus + FOS, Each capsule contains 10⁹ CFU. |
|  |  | Robabeh Soleimani 2023 | 28 | 10 | 33.1 | 27 | 18 | 50.3 | 12 weeks | L. acidophilus, L. casei, L. bulgaricus, L. rhamnosus, B. longum, B. breve, S. salivarius, 9×10⁹ CFU/g |
| 6 | TC | (A)Jing Huang 2022 | 39 | 0.29 | 0.48 | 37 | 0.38 | 0.65 | 12 weeks | Bifidobacterium, Lactobacillus, Enterococcus (Bifico), 840 mg |
|  |  | Aida Mohammadi 2024 | 35 | -18.48 | 37.88 | 35 | 2.78 | 44.91 | 12 weeks | Lactobacillus acidophilus, L. rhamnosus, L. reuteri, L. paracasei, Bifidobacterium longum, Bacillus coagulans,2×10⁹ CFU |
|  |  | Alfonso Sevillano-Jiménez 2022 | 23 | -19.7 | 56.04 | 21 | -2.2 | 44.67 | 6 months | NA |
|  |  | Amir Ghaderi 2019 | 30 | -4.9 | 15 | 30 | 5.9 | 19.5 | 12 weeks | L. acidophilus, B. bifidum, L. reuteri, L. fermentum 8×10⁹ CFU/day |
|  |  | Hamidreza Jamilian 2021 | 25 | -1.6 | 31.69 | 26 | 3.7 | 39.69 | 12 weeks | L. acidophilus, B. lactis, B. bifidum, B. longum, 8×10⁹ CFU/day |
|  |  | Poorya Basafa-Roodi 2024 | 27 | -5.41 | 41.36 | 28 | 0.79 | 30.19 | 8 weeks | L. rhamnosus, L. casei, L. acidophilus, L. bulgaricus, L. plantarum, L. gasseri, L. helveticus, B. lactis, B. breve, B. longum, B. bifidum, S. thermophilus + FOS, Each capsule contains 10⁹ CFU. |
|  |  | Robabeh Soleimani 2023 | 28 | 6.2 | 25.65 | 27 | 23.7 | 28.55 | 12 weeks | L. acidophilus, L. casei, L. bulgaricus, L. rhamnosus, B. longum, B. breve, S. salivarius, 9×10⁹ CFU/g |
| 7 | HDL-cholesterol | (A)Jing Huang 2022 | 39 | -0.11 | 0.20 | 37 | -0.14 | 0.27 | 12 weeks | Bifidobacterium, Lactobacillus, Enterococcus (Bifico), 840 mg |
|  |  | Aida Mohammadi 2024 | 35 | 1.22 | 7.50 | 35 | -5 | 9.89 | 12 weeks | Lactobacillus acidophilus, L. rhamnosus, L. reuteri, L. paracasei, Bifidobacterium longum, Bacillus coagulans,2×10⁹ CFU |
|  |  | Alfonso Sevillano-Jiménez 2022 | 23 | 2.7 | 21.57 | 21 | -1.8 | 15.25 | 6 months | NA |
|  |  | Amir Ghaderi 2019 | 30 | -0.3 | 8.62 | 30 | -1.1 | 6.16 | 12 weeks | L. acidophilus, B. bifidum, L. reuteri, L. fermentum 8×10⁹ CFU/day |
|  |  | Hamidreza Jamilian 2021 | 25 | -1.6 | 8.05 | 26 | -0.6 | 6.36 | 12 weeks | L. acidophilus, B. lactis, B. bifidum, B. longum, 8×10⁹ CFU/day |
|  |  | Poorya Basafa-Roodi 2024 | 27 | -6 | 35.39 | 28 | 3 | 26.65 | 8 weeks | L. rhamnosus, L. casei, L. acidophilus, L. bulgaricus, L. plantarum, L. gasseri, L. helveticus, B. lactis, B. breve, B. longum, B. bifidum, S. thermophilus + FOS, Each capsule contains 10⁹ CFU. |
| 8 | LDL-cholesterol | (A)Jing Huang 2022 | 39 | 0.41 | 0.51 | 37 | 0.44 | 0.64 | 12 weeks | Bifidobacterium, Lactobacillus, Enterococcus (Bifico), 840 mg |
|  |  | Aida Mohammadi 2024 | 35 | -8.74 | 28.22 | 35 | 0.71 | 28.90 | 12 weeks | Lactobacillus acidophilus, L. rhamnosus, L. reuteri, L. paracasei, Bifidobacterium longum, Bacillus coagulans,2×10⁹ CFU |
|  |  | Alfonso Sevillano-Jiménez 2022 | 23 | -3.7 | 37.85 | 21 | -3.5 | 33.75 | 6 months | NA |
|  |  | Amir Ghaderi 2019 | 30 | -3.1 | 29.76 | 30 | 5 | 33.87 | 12 weeks | L. acidophilus, B. bifidum, L. reuteri, L. fermentum 8×10⁹ CFU/day |
|  |  | Hamidreza Jamilian 2021 | 25 | -1.3 | 31.68 | 26 | 3.7 | 36.13 | 12 weeks | L. acidophilus, B. lactis, B. bifidum, B. longum, 8×10⁹ CFU/day |
|  |  | Poorya Basafa-Roodi 2024 | 27 | -7.07 | 26.20 | 28 | 6.64 | 29.29 | 8 weeks | L. rhamnosus, L. casei, L. acidophilus, L. bulgaricus, L. plantarum, L. gasseri, L. helveticus, B. lactis, B. breve, B. longum, B. bifidum, S. thermophilus + FOS, Each capsule contains 10⁹ CFU. |
| 9 | HOMA-IR | Amir Ghaderi 2019 | 30 | -0.8 | 0.85 | 30 | 0.1 | 0.62 | 12 weeks | L. acidophilus, B. bifidum, L. reuteri, L. fermentum 8×10⁹ CFU/day |
|  |  | Hamidreza Jamilian 2021 | 25 | -0.5 | 0.62 | 26 | -0.1 | 0.66 | 12 weeks | L. acidophilus, B. lactis, B. bifidum, B. longum, 8×10⁹ CFU/day |
|  |  | Poorya Basafa-Roodi 2024 | 27 | -0.7 | 3.52 | 28 | -0.3 | 4.36 | 8 weeks | L. rhamnosus, L. casei, L. acidophilus, L. bulgaricus, L. plantarum, L. gasseri, L. helveticus, B. lactis, B. breve, B. longum, B. bifidum, S. thermophilus + FOS, Each capsule contains 10⁹ CFU. |
| 10 | QUICKI | Amir Ghaderi 2019 | 30 | 0.02 | 0.02 | 30 | 0 | 0.01 | 12 weeks | L. acidophilus, B. bifidum, L. reuteri, L. fermentum 8×10⁹ CFU/day |
|  |  | Hamidreza Jamilian 2021 | 25 | 0.01 | 0.01 | 26 | 0 | 0.01 | 12 weeks | L. acidophilus, B. lactis, B. bifidum, B. longum, 8×10⁹ CFU/day |
|  |  | Poorya Basafa-Roodi 2024 | 27 | 0.03 | 0.22 | 28 | 0.04 | 0.26 | 8 weeks | L. rhamnosus, L. casei, L. acidophilus, L. bulgaricus, L. plantarum, L. gasseri, L. helveticus, B. lactis, B. breve, B. longum, B. bifidum, S. thermophilus + FOS, Each capsule contains 10⁹ CFU. |
| 11 | BW | (A)Jing Huang 2022 | 39 | 7.39 | 4.45 | 37 | 7.91 | 4.23 | 12 weeks | Bifidobacterium, Lactobacillus, Enterococcus (Bifico), 840 mg |
|  |  | Alfonso Sevillano-Jiménez 2022 | 23 | -4.4 | 15.5 | 21 | -0.8 | 17.9 | 6 months | NA |
|  |  | Amir Ghaderi 2019 | 30 | 0.2 | 1.2 | 30 | 0.04 | 0.7 | 12 weeks | L. acidophilus, B. bifidum, L. reuteri, L. fermentum 8×10⁹ CFU/day |
|  |  | Edy Husnul Mujahid 2022 | 21 | 0.1 | 11.3 | 21 | -0.2 | 8.25 | 6 weeks | NA |
|  |  | Hamidreza Jamilian 2021 | 25 | 0.5 | 1.8 | 26 | 0.2 | 0.8 | 12 weeks | L. acidophilus, B. lactis, B. bifidum, B. longum, 8×10⁹ CFU/day |
|  |  | Poorya Basafa-Roodi 2024 | 27 | -1.13 | 11.3 | 28 | 1.75 | 14.3 | 8 weeks | L. rhamnosus, L. casei, L. acidophilus, L. bulgaricus, L. plantarum, L. gasseri, L. helveticus, B. lactis, B. breve, B. longum, B. bifidum, S. thermophilus + FOS, Each capsule contains 10⁹ CFU. |
|  |  | Ye Yang 2021 | 33 | 7.4 | 4.4 | 34 | 7.9 | 4.2 | 12 weeks | Bifidobacterium, Lactobacillus, and Enterococcus capsules, 1×10⁷ CFU |
| 12 | BMI | (A)Jing Huang 2022 | 39 | 2.88 | 1.77 | 37 | 3.05 | 1.71 | 12 weeks | Bifidobacterium, Lactobacillus, Enterococcus (Bifico), 840 mg |
|  |  | Aida Mohammadi 2024 | 35 | 0.27 | 5.1 | 35 | 0.54 | 6.6 | 12 weeks | Lactobacillus acidophilus, L. rhamnosus, L. reuteri, L. paracasei, Bifidobacterium longum, Bacillus coagulans,2×10⁹ CFU |
|  |  | Alfonso Sevillano-Jiménez 2022 | 23 | -1.6 | 4.6 | 21 | 0.3 | 5.3 | 6 months | NA |
|  |  | Amir Ghaderi 2019 | 30 | 0.1 | 0.4 | 30 | 0.01 | 0.2 | 12 weeks | L. acidophilus, B. bifidum, L. reuteri, L. fermentum 8×10⁹ CFU/day |
|  |  | Edy Husnul Mujahid 2022 | 21 | 0.02 | 4.41 | 21 | -0.08 | 3.16 | 6 weeks | NA |
|  |  | Hamidreza Jamilian 2021 | 25 | 0.2 | 0.6 | 26 | 0.1 | 0.3 | 12 weeks | L. acidophilus, B. lactis, B. bifidum, B. longum, 8×10⁹ CFU/day |
|  |  | Poorya Basafa-Roodi 2024 | 27 | -0.44 | 4.4 | 28 | 0.57 | 5.7 | 8 weeks | L. rhamnosus, L. casei, L. acidophilus, L. bulgaricus, L. plantarum, L. gasseri, L. helveticus, B. lactis, B. breve, B. longum, B. bifidum, S. thermophilus + FOS, Each capsule contains 10⁹ CFU. |
|  |  | Robabeh Soleimani 2023 | 28 | -1.8 | 2.7 | 27 | -0.8 | 3.0 | 12 weeks | L. acidophilus, L. casei, L. bulgaricus, L. rhamnosus, B. longum, B. breve, S. salivarius, 9×10⁹ CFU/g |
|  |  | Ye Yang 2021 | 33 | 2.9 | 1.8 | 34 | 3 | 1.7 | 12 weeks | Bifidobacterium, Lactobacillus, and Enterococcus capsules, 1×10⁷ CFU |
