# Supplementary material for: Effects of gut microbiota interventions on patients with schizophrenia: a systematic review and meta-analysis
Source: Front Microbiol. 2025 Nov 6;16:1681559. doi: 10.3389/fmicb.2025.1681559 (PMC12630112; doi:10.3389/fmicb.2025.1681559)
Supplement: Supplementary file 4 [file Table_4.DOCX]

**Supplement Table S4: Rating evaluation of GRADE**

| Outcomes | No. of RCTs (studies) | Effect size (95% CI) quality assessment | Quality assessment | | | | | Quality of evidence |
| --- | --- | --- | --- | --- | --- | --- | --- | --- |
|  |  |  | Risk of bias | Inconsistency | Indirectness | Imprecision | Publication bias |  |
| PANSS | 10 (7 studies) | MD = -5.38, 95% CI [-8.7, -2.06] | Serious(-1) | Serious(-1) | no | Serious(-1) | undetected | VERY LOW |
| Negative PANSS | 10 (3 studies) | MD = -1.03, 95% CI [-2.03, -0.04] | Serious(-1) | no | no | no | undetected | MODERATE |
| Positive PANSS | 10 (3 studies) | MD = -0.76, 95% CI [-1.78, 0.27] | Serious(-1) | no | no | Serious(-1) | undetected | LOW |
| BPRS | 10 (3 studies) | MD = -1.68, 95% CI [-4.54, 1.17] | no | Serious(-1) | no | Serious(-1) | undetected | LOW |
| FBS | 10 (7 studies) | MD = -0.36, 95% CI [-0.56, -0.17] | Serious(-1) | no | Serious(-1) | Very serious(-2) | undetected | VERY LOW |
| INS | 10 (5 studies) | MD = -0.36, 95% CI [-1.78, 0.27] | Serious(-1) | no | Serious(-1) | Very serious(-2) | undetected | VERY LOW |
| TG | 10 (7 studies) | MD = -0.25, 95% CI [-0.44, -0.06] | Serious(-1) | Serious(-1) | no | Serious(-1) | undetected | VERY LOW |
| TC | 10 (7 studies) | MD = -0.36, 95% CI [-0.56, -0.17] | no | Serious(-1) | no | Serious(-1) | undetected | LOW |
| HDL-cholesterol | 10 (6 studies) | MD = 0.13, 95% CI [-0.14, 0.41] | Serious(-1) | no | no | Serious(-1) | undetected | LOW |
| LDL-cholesterol | 10 (6 studies) | MD = -0.16, 95% CI [-0.35, 0.04] | Serious(-1) | Serious(-1) | no | Serious(-1) | undetected | VERY LOW |
| HOMA-IR | 10 (3 studies) | MD = -0.63, 95% CI [-0.88, -0.37] | Serious(-1) | no | Serious(-1) | Very serious(-2) | undetected | VERY LOW |
| QUICKI | 10 (3 studies) | MD = 0.01, 95% CI [0.01, 0.02] | Serious(-1) | no | Serious(-1) | Very serious(-2) | undetected | VERY LOW |
| BW | 10 (7 studies) | MD = -0.06, 95% CI [-0.24, 0.13] | Serious(-1) | Serious(-1) | Serious(-1) | Serious(-1) | undetected | VERY LOW |
| BMI | 10 (9 studies) | MD = -0.07, 95% CI [-0.23, 0.09] | Serious(-1) | Serious(-1) | no | Serious(-1) | undetected | VERY LOW |
