# Supplementary material for: Effects of gut microbiota interventions on patients with schizophrenia: a systematic review and meta-analysis
Source: Front Microbiol. 2025 Nov 6;16:1681559. doi: 10.3389/fmicb.2025.1681559 (PMC12630112; doi:10.3389/fmicb.2025.1681559)
Supplement: Supplementary file 5 [file Table_5.DOCX]

**Supplement Table S5: Characteristics of the Studies Included in the Meta-Analysis**

| First Author | Country | Year | Population | Age（mean+SD） | Total/male/female | Intervention | Control | Outcome |
| --- | --- | --- | --- | --- | --- | --- | --- | --- |
| Amir Ghaderi | Iran | 2019 | chronic schizophrenia | T:44.8(8.3) C:43.2(6.0) | T:30/28/2 C:30/28/2 | Probiotic [containing *Lactobacillus acidophilus, Bifidobacterium bifidum, Lactobacillus reuteri, and Lactobacillus fermentum* (8×10⁹ CFU/day )] and Vitamin D (350,000 IU/2 weeks) co-supplementation  Length of Intervention: 12 weeks Freq: every day | Placebo | PANSS; BPRS; FBS; INS; TG; TC; HDL-cholesterol; LDL-cholesterol; HOMA-IR; QUICKI; BW; BMI |
| Ye Yang | China | 2021 | schizophrenia and schizoaffective | T:24.12(5.49) C:23.64(4.99) | T:33/9/24 C:34/12/22 | Probiotic supplements (live combined Bifidobacterium, Lactobacillus, and Enterococcus capsules, 1×10⁷ CFU) Length of Intervention: 12 weeks Freq: 3 times/day | Olanzapine | PANSS; BW; BMI |
| Edy Husnul Mujahid | Indonesia | 2022 | schizophrenia | T:33.57(8.07) C:35.71(6.41) | T:21/16/5 C:21/16/5 | Probiotic supplements  Length of Intervention: 6 weeks Freq: every 12 hours | Placebo | PANSS; BMI; BW |
| Faith B Dickerson | USA | 2014 | schizophrenia | T:44.1(11.0） C:48.1(9.4) | T:33/23/10 C:32/19/13 | Probiotic supplements (Lactobacillus rhamnosus GG + Bifidobacterium animalis subsp. lactis Bb12, each 10⁹ CFU/day)  Length of Intervention: 14 weeks Freq: NA | Placebo | PANSS |
| Poorya Basafa-Roodi | Iran | 2024 | schizophrenia | T:42.19(11.45) C:44.54(9.47) | T:27/14/13 C:28/19/9 | Synbiotic supplement (containing 10^9^ CFU L. rhamnosus, L.casei, L. acidophilus, L. bulgaricus, L. plantarum, L. gasseri, L. helveticus, B. lactis, B. breve, B. longum, B. bifidum, S. thermophilus, and 21 mg fructooligosaccharide) Length of Intervention: 8 weeks Freq: 2 capsules/day | Placebo | FBS; INS; FBS; TG; TC; HDL-cholesterol; LDL-cholesterol; HOMA-IR; QUICKI; BW; BMI |
| Aida Mohammadi | Iran | 2024 | schizophrenia | T:50.29(9.61) C:52.02(8.32) | T:35/25/10 C:35/25/10 | Probiotic supplements [containing Lactobacillus acidophilus, Lactobacillus rhamnosus, Lactobacillus reuteri, Lactobacillus paracasei Bifidobacterium longum, Bacillus coagulans (2×10^9^ CFU)]  Length of Intervention: 12 weeks Freq: 1 capsule/day | Placebo | PANSS; FBS; TG; TC HDL-cholesterol; LDL-cholesterol; BMI |
| Robabeh Soleimani | Iran | 2023 | schizophrenia | T:34.2(10.2) C:36.5(10.8) | T:31/20/11 C:31/19/12 | Probiotic supplements (Lactobacillus Acidophilus, Lactobacillus Casei, Lactobacillus Delbrueckii Subsp. Lactobacillus Bulgaricus, and Lactobacillus Rhamnosus, 9×10⁹ CFU/g) Length of Intervention: 12 weeks Freq: once a day | Placebo | PANSS; BPRS; FBS; INS; TG; TC; FBS; BMI |
| Jing Huang | China, USA | 2022 | schizophrenia | T:24.82(5.64） C:23.43(4.89) | T:39/28/11 C:37/24/13 | Probiotics supplements (containing live *Bifidobacterium, Lactobacillus, and Enterococcus* at concentrations, 840 mg) Length of Intervention: 12 weeks Freq: 840 mg twice daily | Olanzapine | FBS; INS; TG; TC; HDL-cholesterol; LDL-cholesterol; BW; BMI |
| Hamidreza Jamilian | Iran | 2021 | schizophrenia | T:43.9(6.9) C:46.4(10.5) | T:30/NA/NA C:30/NA/NA | Probiotic [containing *Lactobacillus acidophilus, Bifidobacterium lactis, Bifidobacterium bifidum, and Bifidobacterium longum* (8×10⁹ CFU/day)] and selenium (200 μg/day) co-supplementation Length of Intervention: 12 weeks Freq: 8 × 109 CFU/day probiotic supplements and 200 μg/day selenium | Placebo | PANSS; BPRS; FBS; INS; TG; TC; HDL-cholesterol; LDL-cholesterol; HOMA-IR; QUICKI; BW; BMI |
| Alfonso Sevillano-Jiménez | Spain | 2022 | schizophrenia | NA T: 49.2(11.9) M: 50.7(10.1) W: 45(115.5) | T:23/NA/NA C:21/NA/NA | Prebiotics and probiotics dietary advice  Length of Intervention: 6 months Freq: every 15 days (nutrition edu- cation program） | Conventional dietary advice | FBS; TG; TC HDL-cholesterol; LDL-cholesterol; BW; BMI |

Note: PANSS: Positive and Negative Syndrome Scale, BPRS: Brief Psychiatric Rating Scale, FBS: Fasting Blood Sugar, INS: Insulin, TG: Triglyceride, TC: Total Cholesterol, HDL-cholesterol: high density lipoprotein cholesterol, LDL-cholesterol: low density lipoprotein cholesterol, HOMA-IR: homeostasis model assessment of insulin resistance, QUICKI: Quantitative Insulin Sensitivity Check Index, BW: Body Weight, BMI: Body Mass Index, NA: unavailable, Freq: frequency.
